# Networks of Neurodevelopmental Traits, Socioenvironmental Factors, Emotional Dysregulation in Childhood, and Depressive Symptoms Across Development in Two U.K. Cohorts

**DOI:** 10.1176/appi.ajp.20220868

**Published:** 2023-08-16

**Authors:** Luis C. Farhat, Rachel Blakey, George Davey Smith, André Fujita, Elizabeth Shephard, Evie Stergiakouli, Thalia C. Eley, Anita Thapar, Guilherme V. Polanczyk

**Affiliations:** 1 Department of Psychiatry, Faculdade de Medicina FMUSP, Universidade de São Paulo, São Paulo, BR; 2 MRC Integrative Epidemiology Unit, University of Bristol, Bristol, UK; 3 Population Health Sciences, Bristol Medical School, University of Bristol, Bristol, UK; 4 Departamento de Ciência da Computação, Instituto de Matemática e Estatística, Universidade de São Paulo, São Paulo, BR; 5 Department of Child & Adolescent Psychiatry, Institute of Psychiatry, Psychology, & Neuroscience, King's College London, London, UK; 6 Social, Genetic, and Developmental Psychiatry Centre, Institute of Psychiatry, Psychology, and Neuroscience, King's College London, London, UK; 7 Wolfson Centre for Young People’s Mental Health Cardiff University School of Medicine, Cardiff, UK; 8 Child and Adolescent Psychiatry Section, Division of Psychological Medicine, Cardiff University School of Medicine, Cardiff, UK

## Abstract

**Objective:**

Previous population-based studies have identified associations between childhood neurodevelopmental traits and later depression. However, neurodevelopmental traits are highly correlated, which could confound associations when traits are examined in isolation. The authors sought to identify the unique associations between multiple neurodevelopmental traits in childhood and depressive symptoms over development while also considering co-occurring difficulties in multivariate analyses.

**Methods:**

Data from two UK population-based cohorts, the Twins Early Development Study (N = 4,407 independent twins) and the Avon Longitudinal Study of Parents And Children (N = 10,351), were independently analyzed. Bayesian Gaussian graphical models were estimated to investigate pairwise conditional associations between neurodevelopmental traits (autistic, ADHD symptoms; general cognitive, learning, communication abilities), social-environmental stressors (academic performance, peer relations), emotional dysregulation in childhood (7-11 years) and depressive symptoms across development (12, 16, and 21 years).

**Results:**

In both cohorts, there were several unadjusted associations between neurodevelopmental traits and depressive symptoms over development. However, these pairs of variables were mostly found conditionally independent, and none were conditionally associated, after accounting for social-environmental stressors, emotional dysregulation. In turn, social-environmental stressors and emotional dysregulation were conditionally associated with both neurodevelopmental traits and depressive symptoms. Based on replicated data across cohorts, neurodevelopmental traits in childhood could only be indirectly associated with depressive symptoms over development.

**Conclusions:**

This study indicates that associations between childhood neurodevelopmental traits and depressive symptoms over development could be explained by social-environmental stressors and emotional dysregulation. The present findings could inform future research aimed at the prevention of depression in youth with neurodevelopmental disorders.

## Introduction

“Neurodevelopmental disorders” (NDDs) denotes a group of psychiatric diagnoses with early-onset in development, functional impairments across the lifespan, and male predominance (1). Children with NDDs often do not outgrow their difficulties and are at increased risk of developing other psychiatric conditions, such as depressive disorders (henceforth, depression) (2). For example, children with attention-deficit/hyperactivity disorder (ADHD) have ~2 times higher risk for depression over development in comparison to typically developing peers (3–6). Importantly, depression in the context of ADHD may onset earlier (7, 8), recur more often (9), and possibly have worse severity (10, 11). Given the impact of depression in the context of NDDs, prevention is a priority (2). To optimally inform research and interventions aimed at the prevention of depression in youth with NDDs, the factors that are uniquely associated with depression in this population must be identified.

NDDs may be understood as extreme ends of continuous dimensions (1), and thus examining neurodevelopmental traits in samples from the general population may contribute to our understanding about NDDs and the development of depression in their context. Previous population-based studies have corroborated associations between neurodevelopmental traits and the emergence of depression, but most of these studies have concentrated on specific traits in isolation, for example ADHD (12, 13) or autism spectrum disorder (ASD, henceforth autistic) (14, 15) symptoms. Yet, evidence suggests that such traits are highly correlated with each other (1) and with other co-occurring difficulties, including emotional dysregulation, relationship problems, and poor academic competence, which could explain the associations between neurodevelopmental traits and depression (2). Hence, focusing exclusively on individual traits separately without accounting for the simultaneous effects of others nor highly correlated co-occurring difficulties and stressors has limited capacity to clarify their unique associations over development. Consequently, whether the findings reported by previous research represent specific associations between certain neurodevelopmental traits in childhood and depression later in life, or are confounded by highly correlated factors, is unclear.

The current study aimed at filling this gap. We used data from two UK population-based cohorts and estimated gaussian graphical models (GGMs) to identify the unique associations between neurodevelopmental traits (autistic and ADHD symptoms; general cognitive, learning, and communication abilities), social-environmental stressors (peer relationships, academic competence), emotional dysregulation in childhood (ages 7-11 years), and depressive symptoms over development (ages 12, 16, and 21 years). GGMs are probabilistic network models that allow all variables to co-vary and result in partial correlations between any two given variables that represent conditionally dependent relationships controlled for the effects of all other variables in the model (16). There is a close relationship between partial correlations and coefficients from multiple regression models, and GGMs may be understood as linking separate multiple regression models where each variable is regressed against the other variables in the network (17). Hence, GGMs are powerful data-driven tools to map out multicollinearity and predictive mediation (17) and may provide a clearer perspective to complex patterns of associations such as those that exist between neurodevelopmental traits, social-environmental stressors, and emotional dysregulation in childhood and depressive symptoms over development.

We hypothesized that: (1) neurodevelopmental traits would be associated with each other; (2) ADHD symptoms would be associated with depressive symptoms after accounting for other neurodevelopmental traits, social-environmental stressors, and emotional dysregulation; (3) any association between other neurodevelopmental traits and depressive symptoms would be explained by ADHD symptoms, social-environmental stressors, or emotional dysregulation. We hypothesized that ADHD symptoms would be uniquely associated with depressive symptoms because of their shared etiological factors (e.g., high genetic correlation) (18).

## Methods

We pre-registered our study in Open Science Framework (OSF) (https://doi.org/10.17605/OSF.IO/QP7ZM). For a copy of the pre-registration file and *post hoc* changes, with reasons, see Supplement A in the online supplement.

### Participants and setting

We analyzed data from the Twins Early Development Study (TEDS) and the Avon Longitudinal Study of Parents And Children (ALSPAC), two well-established longitudinal studies over development. For details about both cohorts, see Supplement B in the online supplement. For TEDS, as many as 5,554 families (11,108 individuals) were invited to participate in all waves of data collection of interest to the current analyses. For ALSPAC, the total sample size comprises 14,901 children who were alive at 1 year of age.

### Variables, data sources, and measurements

We included measures of multiple neurodevelopmental traits, two social-environmental stressors, and emotional dysregulation in childhood (ages 7-11 years), and depressive symptoms over development (ages 12, 16, and 21 years) (Table 1). For a complete description of variables, measures, and scoring rules, see Supplement C in the online supplement.

We selected variables considering theoretical and operational aspects of NDDs and their association with depression, previous research, and availability of measurements in the cohorts. We only selected variables if data for that domain were available to us from both TEDS and ALSPAC. For neurodevelopmental traits, our choice of domains was informed by the approach of DSM-5, which groups ADHD, ASD, intellectual disability, communication disorders, and specific learning disorders as NDDs. The selected measures and instruments were similar to those from previous studies (19, 20). For social-environmental stressors, our choice of domains was informed by the fact that children with NDDs often experience more difficulties in peer relationships and schoolwork than typically developing children, which may contribute to the development of depression (2, 21). Additionally, previous work has suggested that peer relationships and academic competence may mediate the association between specific neurodevelopmental traits and depression (13, 22), although conflicting findings have also been described (23). For emotional dysregulation, the instrument used is composed of items included in the emotional, hyperactivity, and conduct scales of the Strengths and Difficulties Questionnaire (SDQ) (24), and therefore may have some overlap with the measures of depression and ADHD included in this study.

For each variable, we adopted prorated continuous scores. Although individual items have been more widely used in GGMs, other studies (25) also employed scores instead of items given some advantages of the former, such as decreased estimation problems (26) and increased interpretability.

### Statistical analyses

We conducted analyses for each cohort separately. We excluded individuals who had all neurodevelopmental trait data missing. For TEDS, we also excluded individuals following the cohort’s standard exclusion routines (see Supplement D in the online supplement). Our TEDS and ALSPAC analyses included 4,407 and 10,351 individuals, respectively.

We adopted multiple imputation of 50 datasets with the R package *mice* (27) for individuals who provided data on at least one neurodevelopmental trait. For the proportion of missing data for each variable, see Supplement E in the online supplement. We also applied the shrunken empirical cumulative distribution function to conduct non-paranormal transformation of variables with the R package *huge* (function “*huge.npn*”) (28) because our variables had skewed distributions.

We calculated Pearson correlation coefficients *r* to estimate zero-order correlations (i.e., unadjusted associations) between neurodevelopmental traits in childhood and depressive symptoms over development. To adjust for multiple testing, we derived a p < 2.38x10^-3^ by applying a Bonferroni correction to the nominal alpha of 0.05 (adjusting for the 21 pairings tested).

To estimate GGMs, we adopted the Bayesian approach (29) with the R package *BGGM* (30). We fit a model for each imputed dataset and pooled their estimates for the primary analysis (function “*bggm_missing*”). We set the prior scale to 0.2 and drew 10,000 samples from the posterior. Initially we estimated GGMs with neurodevelopmental traits alone and then we estimated GGMs with all variables. Visual inspection of trace plots demonstrated acceptable mixing of chains and are available in OSF (https://doi.org/10.17605/OSF.IO/P7WMK).

We quantified the support in the data for two competing, complementary hypotheses for each partial correlation (*ρ*) (*ℋ*_1_: *ρ* ≠ 0; *ℋ*_0_: *ρ* = 0) through the Bayes factor (BF) (31). To determine if two variables were conditionally independent given the other variables (i.e., *ρ* = 0), we calculated BF_01_ and used BF_01_ ≥ 3 as the threshold to determine that there was sufficient evidence to accept *ℋ*_1_: *ρ* ≠ 0. To determine if two variables were associated with each other after accounting for the remaining variables (i.e., *ρ* ≠ 0), we calculated the reciprocal BF_10_ and used BF_10_ ≥ 3 as the threshold to determine that there was sufficient evidence to accept *ℋ*_1_: *ρ* ≠ 0. When both BF_10_ < 3 and BF_01_ < 3, we classified the partial correlations as ‘ambiguous’. An equivalent approach would be to calculate BF_10_ only and consider BF_10_ ≥ 3 as sufficient evidence for *ℋ*_1_; BF_10_ ≤ 0.33 as sufficient evidence for *ℋ*_0_; and 0.33 < BF_10_ < 3 as insufficient evidence (ambiguous). We opted for a BF threshold of 3 because it has been demonstrated to return desirable asymptotic properties in a simulation study (32). However, because the BF is a continuous metric and higher values indicate stronger evidence (33), we also reported BF values to inform about the strength of evidence in favor of *ℋ*_1_ or *ℋ*_0_ for each pair of variables (see supplement F in the online supplement).

We only drew inferences on findings that were replicated in TEDS and ALSPAC. We calculated the network density (the number of estimated conditional associations relative to the total number of possible conditional associations) to provide an indication of how well connected the variables were. For conditional associations for which we accepted *ℋ*_1_: *ρ* ≠ 0, we estimated the magnitude of *ρ* by extracting the posterior mean with 95% credible intervals (CrI). We compared the relative magnitude of the replicated *ρ* by computing *r* of the weighted adjacency matrices of the two cohorts. To provide a graphical illustration of the GGMs, we plotted networks based on the Fruchterman-Reingold algorithm with the R package *qgraph* (34) and fixed the average layout to facilitate interpretation. To evaluate whether symptoms would form clusters, we estimated the nature and number of groups of symptoms across 5,000 iterations with the Spinglass algorithm (35). The Spinglass provides both node-level information (which nodes are grouped together) and community-level information (the frequency of community structures across iterations). We converted partial correlations to standardized regression coefficients (*β*) (29). In an attempt to provide effect sizes for the indirect associations, we extrapolated from mediation analyses and calculated a version of the index of mediation *ab_cs_* = *β_MX_* * *β_YM_* (36) with the converted *β* despite the fact that *β_MX_* is also adjusted for *Y* in our GGM analyses. To quantify how well depressive symptoms were predicted by all remaining nodes, we calculated the Bayesian R^2^ (29).

The robustness of our findings was examined in sensitivity analyses performed by increasing (SD = 0.4) and decreasing (SD = 0.1) the prior scale; only including individuals with ≥ 70% complete neurodevelopmental trait data (n = 3,106 for TEDS; n = 7,756 for ALSPAC); adjusting for differences in age at data collection and sex by using the residuals from linear regression models to estimate GGMs.

## Results

### Neurodevelopmental traits

**Figure 1:**
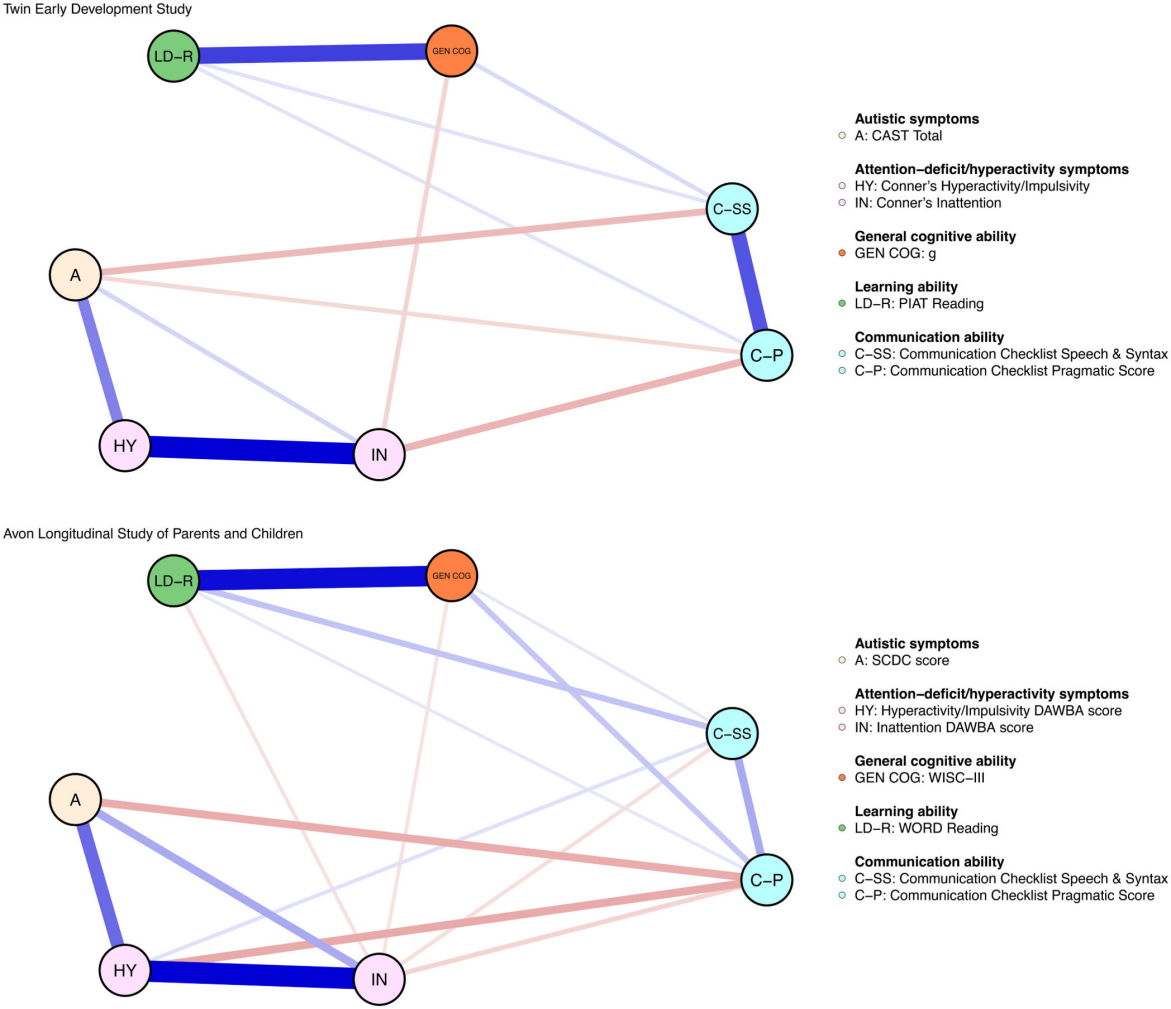
Network plots for neurodevelopmental traits in childhood. The network plot for the Twins Early Development Study is presented at the top and the one for the Avon Longitudinal Study of Parents And Children is presented at the bottom. Variables are represented as nodes (circles) and are colored according to their domain. Edges between two nodes represent partial correlations between two variables. The width of edges is proportional to the strength of the partial correlation. Positive and negative partial correlations were colored in blue and red, respectively.

**Figure 2:**
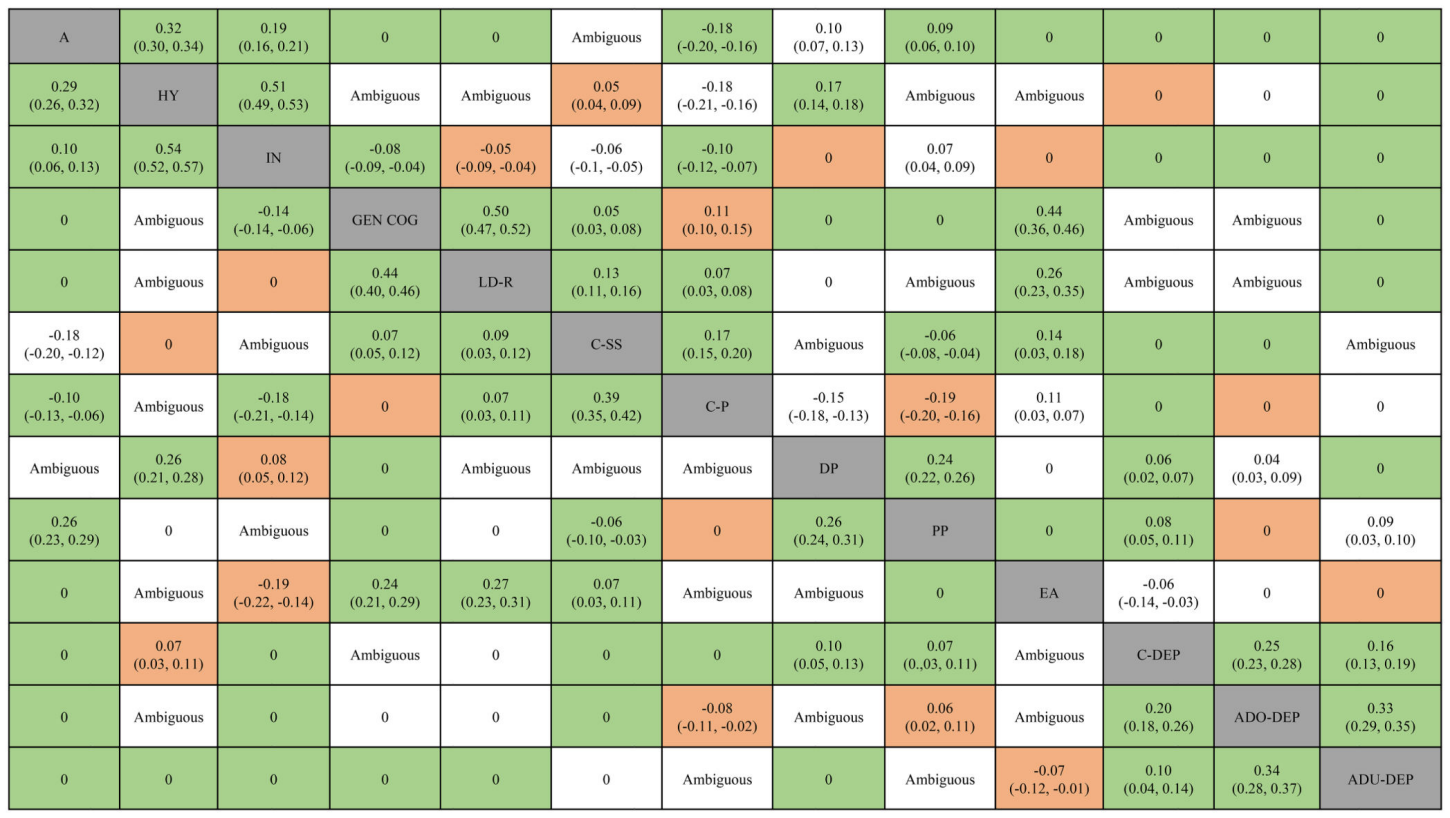
Conditional associations between neurodevelopmental traits, social-environmental stressors, emotional dysregulation in childhood and depressive symptoms over development and their relative magnitude. The values for the Twins Early Development Study are presented in the lower triangle and the ones for the Avon Longitudinal Study of Parents And Children are presented in the top triangle. Values represented are posterior means with 95% credible intervals. Values for the neurodevelopmental traits are from the model with neurodevelopmental traits only. Green indicates results that were replicated in both cohorts, orange indicates results that are discordant between both cohorts, and white indicates results which were ambiguous in at least one of the cohorts. Abbreviations: A = Autistic symptoms; HY = Hyperactivity/impulsivity symptoms; IN = Inattentive symptoms; GEN COG = General cognitive ability; LD-R = Learning ability; C-SS = Communication ability, speech & syntax; C-PP = Communication ability, pragmatic; DP = Emotional dysregulation; PP = Peer problems; EA = Educational achievement; C-DEP = Childhood depressive symptoms; ADO-DEP = Adolescent depressive symptoms; ADU-DEP = Adult depressive symptoms.

Findings from both cohorts supported the presence of 11 (52%) of the 21 possible conditional associations between two neurodevelopmental traits (Figure 1). The relative magnitude of *ρ* for these associations was similar across cohorts (r = 0.93), as was the order of the strongest associations, such as hyperactive/impulsive and inattentive symptoms; general cognitive and learning abilities; autistic and hyperactive/impulsive symptoms (Figure 2). Additionally, analyses of data from both cohorts indicated that only 2 (9.5%) of the 21 pairs of variables were conditionally independent given the other neurodevelopmental traits: autistic symptoms and general cognitive, learning abilities (Figure 2).

### Neurodevelopmental traits and social-environmental stressors, emotional dysregulation

**Figure 3:**
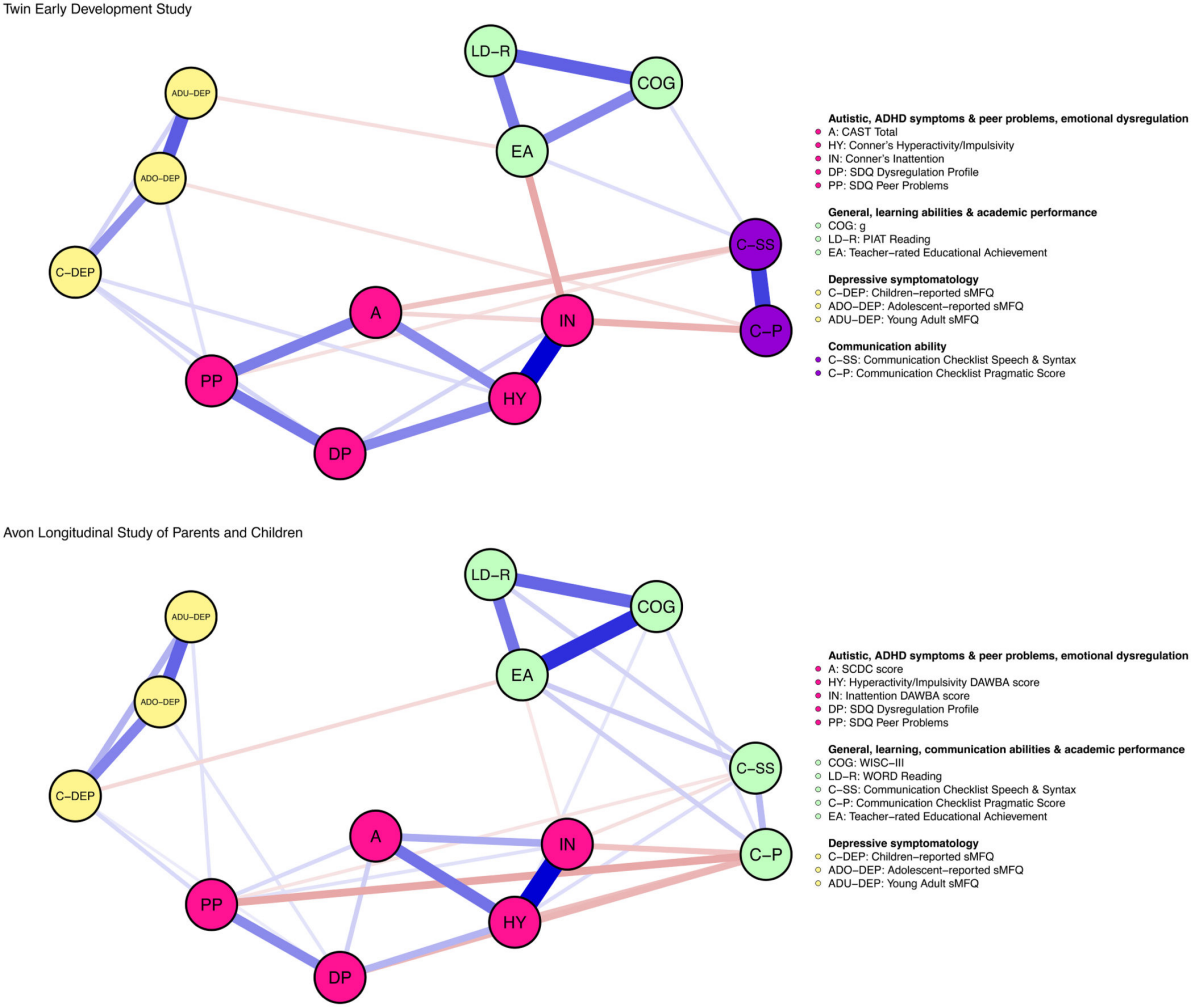
Network plots for neurodevelopmental traits, social-environmental stressors, and emotional dysregulation in childhood and depressive symptoms over development. The network plot for the Twins Early Development Study is presented at the top and the one for the Avon Longitudinal Study of Parents And Children is presented at the bottom. Variables are represented as nodes (circles) and are colored according to their community as identified by the Spinglass algorithm. These exact cluster solutions that are presented in the figure were retrieved in 93% and 88% of the 5,000 iterations for TEDS and ALSPAC, respectively. Edges between two nodes represent partial correlations between two variables. The width of edges is proportional to the strength of the partial correlation. Positive and negative partial correlations were colored in blue and red, respectively.

Findings from both cohorts supported the presence of 6 (29%) of the 21 possible conditional associations between neurodevelopmental traits and social-environmental stressors, emotional dysregulation (Figure 3). The relative magnitude of *ρ* for these associations was similar across cohorts (r = 0.66), as was the order of the strongest associations, such as general cognitive, learning abilities and academic competence; hyperactive/impulsive symptoms and emotional dysregulation (Figure 2). Additionally, analyses of data from both cohorts indicated that 3 (14.5%) of the 21 pairs of variables were conditionally independent given the other variables: general cognitive ability and emotional dysregulation, peer problems; autistic symptoms and academic competence (Figure 2).

### Neurodevelopmental traits and depressive symptoms

There were 12 significant unadjusted associations between neurodevelopmental traits and depressive symptoms over development (see supplement G in the online supplement for zero-order correlations). However, findings from both cohorts did not support the presence of any conditional associations between neurodevelopmental traits and depressive symptoms over development (Figure 3).

Instead, analyses of data from both cohorts indicated that 12 (57%) of these 21 pairs of variables were conditionally independent given the other variables: childhood depressive symptoms and autistic, inattentive symptoms, communication abilities; adolescent depressive symptoms and autistic, inattentive symptoms, speech & syntax communication ability; early adulthood depressive symptoms and autistic, ADHD symptoms, general cognitive, learning abilities (Figure 2). Notably, 9 of the 12 pairs of variables with significant unadjusted associations were found conditionally independent in GGMs.

Community detection algorithm corroborated that depressive symptoms were segregated from neurodevelopmental traits in the network as unique clusters for depressive symptoms were identified in both cohorts (Figure 3). Additionally, a small proportion of the variance of depressive symptoms in childhood, adolescence, and early adulthood was explained by the remaining variables in the model as indexed by Bayesian R^2^ (see supplement H in the online supplement).

### Social-environmental stressors, emotional dysregulation and depressive symptoms

Findings from both cohorts supported the presence of 2 (22%) of 9 possible conditional associations between social-environmental stressors, emotional dysregulation and depressive symptoms: childhood depressive symptoms were associated with emotional dysregulation as well as with peer problems in both cohorts. Additionally, depressive symptoms (either during childhood, adolescence, or early adulthood) were also conditionally associated with academic competence in both cohorts (Figure 3).

Considering the index of mediation (see supplement I in the online supplement), childhood depressive symptoms increased by 0.028 (95% CrI 0.014, 0.045) and 0.011 (95% CrI 0.004, 0.019) standard deviations (SD) for every 1 SD increase in hyperactivity/impulsivity symptoms indirectly via emotional dysregulation in TEDS and ALSPAC, respectively. Similarly, childhood depressive symptoms increased by 0.022 (95% CrI 0.010, 0.037) and 0.007 (95% CrI 0.003, 0.014) SD for every 1 SD increase in autistic symptoms indirectly via peer problems in TEDS and ALSPAC, respectively.

### Sensitivity analyses

The findings did not change considerably in sensitivity analyses as we did not find conditional associations between neurodevelopmental traits and depressive symptoms but continued to find conditional associations between social-environmental stressors, emotional dysregulation and neurodevelopmental traits, depressive symptoms. For a summary of changes in the sensitivity analyses, network plots and tables with the magnitude of *ρ* for all sensitivity analyses, see Supplement J in the online supplement.

## Discussion

In this study, we evaluated the unique associations between neurodevelopmental traits, social-environmental stressors, emotional dysregulation in childhood and depressive symptoms over development using data from two well-established UK longitudinal studies over development. Through the adoption of a Bayesian approach, we were able to classify whether there was sufficient evidence in favor of conditional association (i.e., non-zero partial correlation), conditional independence (i.e., partial correlation of zero) or insufficient evidence (i.e., ‘ambiguous’) for every pair of variables. Findings from both cohorts supported the presence of numerous conditional associations between two neurodevelopmental traits. There were several significant unadjusted associations (based on zero-order correlations) between neurodevelopmental traits and depressive symptoms over development; however, most of these pairs of variables with significant unadjusted associations were found conditionally independent, and none were conditionally associated, after accounting for social-environmental stressors, emotional dysregulation. In turn, social-environmental stressors and emotional dysregulation were conditionally associated with both neurodevelopmental traits and depressive symptoms, particularly during childhood. Based on replicated data across cohorts, neurodevelopmental traits in childhood could only be indirectly connected to depressive symptoms over development. Taken together, the present findings indicate that associations between neurodevelopmental traits in childhood and depressive symptoms over development could be explained by social-environmental stressors and emotional dysregulation.

We found numerous conditional associations between two neurodevelopmental traits in childhood, which reinforces the importance of considering multiple neurodevelopmental traits simultaneously, rather than examining them individually, in future studies conducted on samples from the general population. Whether our findings would generalize to clinical samples is unclear, particularly for some of our unexpected findings (e.g., autistic symptoms were independent from general cognitive, learning abilities given the other traits). Because children with NDDs often present with symptoms from multiple NDDs simultaneously (1), it is possible that similar associations would be found in a clinical sample, and future research should examine this question directly. It would be of particular interest to evaluate how neurodevelopmental traits are related to each other in a transdiagnostic sample of individuals diagnosed with NDDs. Despite some consensus over the need to move beyond discrete diagnostic classification (37), there has been relatively little clinical transdiagnostic research in the field.

We found several significant unadjusted associations between neurodevelopmental traits in childhood and depressive symptoms over development, which is in line with previous findings from population-based studies (12-15, 19). However, we expanded on this literature by demonstrating that most of these pairs of variables with significant unadjusted associations were found conditionally independent after accounting for the effects of social-environmental stressors and emotional dysregulation in multivariate analyses. Notably, based on replicated data across cohorts, because neurodevelopmental traits in childhood could only be indirectly connected to depressive symptoms over development, any predictive effect from the former to the latter would be mediated by social-environmental stressors and emotional dysregulation in childhood. These findings suggest that intervening on social-environmental stressors and emotional dysregulation could contribute to the prevention of depression in children and young people with NDDs. Overall, these indirect associations had small effects, but effective prevention efforts for depression will likely need to manage numerous risk factors of small effects (38), including in the context of NDDs. Consistently, in the context of ADHD, there is some preliminary evidence that programs focused on emotional dysregulation and family support may be efficacious in reducing depressive symptoms (39). Additional research should continue to examine this important problem in ADHD and other NDDs.

Our findings provide strong evidence that associations between childhood neurodevelopmental traits and depressive symptoms over development could be explained by social-environmental stressors and emotional dysregulation, which is in line with theories about adult disorders with childhood antecedents (40). However, we cannot definitively rule out that there could be an association between neurodevelopmental traits in childhood and depressive symptoms over development to avoid inferring that absence of evidence equates to evidence of absence. Specifically, there were significant associations in unadjusted analyses but inconclusive evidence in GGMs for three pairs of variables involving neurodevelopmental traits and depressive symptoms for at least one time point. We found insufficient evidence (‘ambiguous’) for associations between hyperactivity/impulsivity symptoms and adolescent depressive symptoms in TEDS, and we found discordant findings across TEDS and ALSPAC for associations between hyperactivity/impulsivity symptoms, pragmatic communication ability and childhood, adolescent depressive symptoms, respectively. GGMs require large sample sizes to identify multiple small effects simultaneously, and even though our analyses included thousands of individuals it is expected that some associations would be classified as ‘ambiguous’ or fail to replicate (41, 42). Additional studies are required to address this question definitively.

### Strengths and limitations

The biggest strength of our study was the rigorous methodology that was adopted to increase the confidence in our findings. Specifically, we formally tested the null hypothesis of conditional independence, and we independently analyzed data from two cohorts and conservatively drew inferences for replicated associations only. Although the models were not identical, we were able to identify similar patterns of associations across the two cohorts that corroborated our hypotheses, particularly (1) and (3). GGMs are explorative in nature and ideal tools for hypothesis-generation (43). Our findings generated several hypotheses that should be tested in future research, e.g., using causal models such as directed acyclic graphs in clinical samples, which in turn could contribute to advance further our understanding of the association between NDDs and depression, as well as inform research aimed at the prevention of depression in youth with NDDs.

However, our study also has limitations. First, we were unable to include other important social-environmental stressors such as home chaos and parent-child relationships, among others. Variable selection in GGMs is driven by substantiative considerations (43) and some have argued that researchers should select the minimally complete set of variables to model the phenomena of interest considering the clinical hypothesis (44). In GGMs the associations between variables are dependent on the variables that are included in the model. We expect that including additional social-environmental stressors would strengthen the direction of the findings presented in this study since the inclusion of a few of them already explained most of the unadjusted associations between neurodevelopmental traits and depressive symptoms. Additional research could investigate this question directly if more measurements are available.

Second, there may exist some content overlap across some of the domains included in our analyses, which may have influenced some of our findings. Most notably, the measure of emotional dysregulation is partially composed of items belonging to the emotional and hyperactivity SDQ scales, which may have some overlap with the measures that were included in our study for depressive and ADHD symptoms, respectively. However, empirical data have shown that the Child Behavior Checklist Dysregulation Profile (CBCL-DP), a related measure of emotional dysregulation that is composed of three similar syndrome scales (anxious/depressed, attention problems, aggressive behavior), is distinct from each of its components either alone or in tandem (45). Additionally, the issue of content overlap has been discussed across other domains, e.g., ADHD and autistic symptoms (46), and may underscore a broader limitation of the current conceptualization of psychiatric phenomena, which might be applicable to other trans-diagnostic studies.

Third, it is possible that the associations between depressive symptoms and neurodevelopmental traits would be more robust if a strict phenotype of depression (e.g., major depressive disorder) was considered rather than depressive symptoms only (12). However, we opted to use dimensional scores and avoid dichotomizing based on cut-off scores in rating scales because this has been shown to negatively impact the recovery performance of networks (47).

Fourth, the neurodevelopmental traits were collected at different time points spanning 2-year intervals from 8-10 years (TEDS) and 7-9 years (ALSPAC). Although this is not ideal because neurodevelopmental traits undergo maturational changes, children at these ages are at relatively similar developmental stages.

Lastly, both cohorts are susceptible to non-random attrition. For instance, in ALSPAC, it has been demonstrated that individuals at elevated risk of psychopathology are more likely to drop-out of the study (48). Detailed cohort-level attrition rates have been provided in previous publications (49, 50). However, we used different statistical methods (multiple imputation, completers analyses) to assess the impact of missingness and found similar patterns of results.

## Conclusions

In conclusion, our study adopted rigorous methodology and provided relevant findings for future research in the field of NDDs and depression. Our findings indicate that associations between neurodevelopmental traits in childhood and depressive symptoms over development could be explained by social-environmental stressors and emotional dysregulation. These findings could improve the understanding of the association between NDDs and depression, as well as inform research aimed at the prevention of depression in youth with neurodevelopmental disorders.

## Supplementary Material

Supplement

## Figures and Tables

**Table 1:** Domain, sub-domain, and instruments included in this study stratified by cohort.

Twins Early Development Study							
Domain	Sub-domain	Instrument	Rating scale	Number of items	Score range	Informant	Age at collection
*Neurodevelopmental traits*							
ADHD symptoms	Hyperactivity/impulsivity	CPRS-R^a^	4-point Likert	9	0; 27	Parents	8 years old
	Inattention						
Autistic symptoms	NA	CAST^a^	Binary	31	0; 31		
General cognitive ability	NA	WISC-III^a^/CAT3^a^	NA	85	−3; 3	Self	9 years old
Communication ability	Speech & Syntax	CCC^a^	3-point Likert	8	0; 16	Parents	
	Pragmatic			4	0; 8		
Learning ability	NA	PIAT^a^	NA	82	0; 82	Self	10 years old
*Co-occurring emotional dysregulation and social-environmental stressors*							
Emotional dysregulation	NA	SDQ-DP^a^	3-point Likert	5	0; 10	Parent	9 years old
Peer relationships	NA	SDQ-PP^a^					
Academic competence	NA	KS2 assessment^a^	5-point Likert	9	9; 45	Teacher	10 years old
*Depressive symptoms*							
Depressive symptoms	NA	sMFQ^a^	3-point Likert	13	0; 26	Self	12 years old
							16 years old
				8	0; 16		21 years old
**Avon Longitudinal Study of Parents And Children**							
**Domain**	**Sub-domain**	**Instrument**	**Rating scale**	**Number of items**	**Score range**	**Informant**	**Age at collection**
*Neurodevelopmental traits*							
ADHD symptoms	Hyperactivity/impulsivity	DAWBA^a^	3-point Likert	9	0; 18	Parents	7.58 years old
	Inattention						
Autistic symptoms	NA	SCDC^a^		12	0; 24		
General cognitive ability	NA	WISC-III^a^	NA	NA	45; 151	Self	8.5 years
Communication ability	Speech & Syntax	CCC^a^	3-point Likert	15	45; 70	Parents	9.58 years
	Pragmatic			38	96; 162		
Learning ability	NA	WORD^a^	NA	NA	0; 50	Self	7.5 years
*Co-occurring emotional dysregulation and social-environmental stressors*							
Emotional dysregulation	NA	SDQ-DP^a^	3-point Likert	5	0; 10	Parents	9.58 years
Peer relationships	NA	SDQ-PP^a^					
Academic competence	NA	KS2 point score^a^	NA	NA	0; 99	NA	11 years
*Depressive symptoms*							
Depressive symptoms	NA	sMFQ^a^	3-point Likert	13	0; 26	Self	12 years
							16 years
							21 years

a CAST = childhood autism spectrum test; CAT3 = cognitive abilities test 3; CCC = children’s communication checklist; CPRS-R = DSM-IV ADHD Conner’s parent rating scale-revised; DAWBA = ADHD section of the development well-being assessment; KS2 = Key stage 2; PIAT = Peabody individual achievement test; SCDC = Social and communication disorders checklist; SDQ-DP = Strengths and difficulties questionnaire dysregulation profile; SDQ-PP = Strengths and difficulties questionnaire peer problems; sMFQ = short mood and feelings questionnaire; WISC-III = Wechsler intelligence scale for children-III-UK; WORD = Wechsler objective reading dimensions.
