# Supplementary material for: Networks of Neurodevelopmental Traits, Socioenvironmental Factors, Emotional Dysregulation in Childhood, and Depressive Symptoms Across Development in Two U.K. Cohorts
Source: Am J Psychiatry. Author manuscript; Available in PMC 2024 Feb 23. (PMC7615665; doi:10.1176/appi.ajp.20220868)
Supplement: Supplement [file EMS192757-supplement-Supplement.pdf]

Supplement A. *A priori* registered plan and *post hoc* changes, with reasons.

The plan of analysis below was pre-registered within the usual workflow required by the Twin Early Development Study (TEDS) for data requests (see https://www.teds.ac.uk/researchers/teds-data-access-policy). At the time, we did not expect to be able to use the data from the Avon Longitudinal Study of Parents and Children (ALSPAC). Therefore, some of the deviations from the original plan arose from harmonization across cohorts.

Deviations from the pre-registered plan

- **Variables:** We were not able to incorporate mathematical test scores as an indicator of learning abilities and we only used total autistic symptoms scores instead of the triad of autistic symptoms (social impairments [SIs], communication impairments [CIs], and restricted repetitive behaviors and interests [RRBIs]) (1, 2) because these data were not available to us from ALSPAC. Considering that the associations from gaussian graphical models (GGMs) are conditional on the variables that are included in the model (3), we opted to only include variables which were available from the two cohorts.
- **Main analysis plan:** Our original analysis plan was based on the traditional frequentist approach for psychological network analyses and was heavily influenced by previous studies in the field, e.g., Fried et al., who evaluated in a series of stepwise models whether associations between depression symptoms and inflammation markers survived incorporation of covariates in their models (4). Upon reflection, however, in the traditional frequentist approach there are no guarantees that unselected edges are statistically indistinguishable from zero or that evidence for their absence is strong (3). Therefore, we opted to adopt the Bayesian approach to estimate our models instead because it allows for quantification of the evidence against edge inclusion and hypothesis testing (5, 6). We also did not create stepwise models because adding variables to a GGM increases the sampling variability of a partial correlation, which in turn reduces the chances that the association is detected (7).
- **Sensitivity analyses plan:** We originally proposed to conduct a separate analysis with data from the other random twin from TEDS to replicate findings from the main analysis, also from TEDS. However, because we acquired independent data from a different cohort study (ALSPAC) we opted not to conduct those analyses, which would be based on dependent data and therefore would provide weaker evidence of replicability. We also originally proposed to estimate models with only data from individuals with 'neurodevelopmental difficulties' as described by Eyre et al. (8). However, individuals with neurodevelopmental difficulties would be determined based on cut-off values and there is evidence that this procedure is associated with worse recovery of the network structure and may introduce bias in the analyses (9). Lastly, we planned to conduct a sensitivity analysis including parent-reported depressive symptoms at ages 12 and 16 years. However, because we only had self-reported scores at age 21 years, we opted not to conduct the sensitivity analysis mixing informants, which could be inconclusive.

OSF REGISTRIES Add New My Registrations Help Donate Join Login

Characterizing the network structure of neurodevelopmental difficulties in childhood and its association with depression in adolescence and early adulthood.

Public registration Updates

Overview
Files
Resources
Wiki
Components 0
Links 0
Analytics
Comments 0
Open practice resources
Data
Analytic code
Materials
Papers
Supplements

Study Information

Hypotheses

This proposal has three overarching aims.
1) To evaluate the network structure of neurodevelopmental difficulties in childhood.
2) To evaluate which neurodevelopmental traits across childhood are independently related to depression in adolescence and early adulthood and which ND traits are only associated with depression outcomes via other ND.
3) To assess the role of emotional dysregulation, educational attainment and peer problems in longitudinal associations between ND in childhood and depression in adolescence and adulthood.

We hypothesize that:
1) Given the frequent co-occurrence of ND, the network structure of ND in childhood will be characterized by positive cross-disorder connections (i.e., between symptoms of ADHD, ASD, ID, LD and CD).
2) Given potential shared genetic mechanisms between ADHD and depression (Riglin et al., 2020), we expect a direct association between ADHD and depression, but not between other ND and depression.
3) We expect that all childhood ND will be indirectly related to depression in adolescence/early adulthood through links between emotional dysregulation, academic attainment and peer problems.
4) We expect that the predictability of depression in adolescence/early adulthood will increase once we consider childhood depression/anxiety symptoms/diagnosis in our model.

Design Plan

Study type

Observational Study - Data is collected from study subjects that are not randomly assigned to a treatment. This includes surveys, “natural experiments,” and regression discontinuity designs.

Blinding

No blinding is involved in this study.

Is there any additional blinding in this study?

No response

Study design

Longitudinal design with 5 exposures in childhood and 3 outcomes at later life in adolescence (2 outcomes) and adulthood (1 outcome).

No files selected

Randomization

No response

Sampling Plan

Existing Data

Registration prior to accessing the data

Explanation of existing data

No response

Data collection procedures

Coinciding with the start of the TEDS study, the OPCS (Office of Population, Censuses and Surveys), soon renamed to the ONS (Office for National Statistics), set up a national register of twins, triplets and other multiple births, called the National Twins Plus Register.

Once TEDS had obtained funding from the MRC (Medical Research Council), ONS agreed to ask families of twins to give consent for joining TEDS at the same time as asking them for consent for joining Twins Plus. ONS agreed to do this for all recorded and traceable live twin births in England and Wales between January 1994 and December 1996. Hence the initial contact with families, by ONS, was to invite each family to take part in either or both of TEDS and the Twins Plus Register.

ONS sent each family a pack including the following materials:

An invitation letter (pdf)
A brief information sheet about the TEDS study (pdf)
A leaflet describing the Twins Plus Register (pdf)
A reply card (pdf)
A postage-paid return envelope for the card
The reply card had two tick boxes in which parents could express an interest in the Twins Plus Register and in TEDS respectively; it also had a space in which parents could record address changes. The cards were returned to ONS, not to TEDS.

For those families who ticked the box expressing an interest in TEDS, ONS would record the essential details to be passed on to TEDS: the forename, surname and address of the parent would had been contacted (who would become the "contact parent" in TEDS); the names, birth order and dates of birth of the two twins; and an ID assigned by ONS to the family. Periodically during the recruitment process, ONS would compile these details of newly recruited families into a spreadsheet list. The list would then be saved (usually as a delimited text file) onto a floppy disk, which was then posted to the TEDS office accompanied by a letter. The surviving files sent in this way, and scanned copies of the letters, have now been archived in TEDS.

On receipt of these files, the details of the parents and twins were added to the TEDS admin database. In the TEDS database, each family was assigned a unique ID number (the FamilyID, with 4 or 5 digits) and each twin was additionally assigned a unique ID number (the TwinID, with 7 or 8 digits comprising the FamilyID followed by a 3 further digits). The original ONS family IDs (containing both letters and numbers) were not used further in TEDS except in the event of correspondence with ONS, for example to exchange details of address changes.

Hence, over the course of recruitment and correspondence with ONS, the original TEDS sample of 16,810 families was collected and added to the TEDS admin database. This is sometimes referred to in TEDS as the "ONS sample". These were the 16810 families that formed the sample for the 1st Contact study.

For this study, we will plan to leverage the data already collected by TEDS investigators and made available to bona fide researcher affiliated to a recognised academic research institution.

No files selected

Sample size

The TEDS is composed of four cohorts (Jan-Aug/94; Sep/94-Aug/95;Sep/95-Aug/96; Sep-Dec/96). These cohort groupings were generally made for administrative reasons: from the 7 Year study onwards, twins' teachers were contacted one cohort at a time, during the school year in which the twins reached a given age. These cohort categories are arbitrary in many respects, although in some studies the data collection procedures changed from one cohort to the next, e.g., not all cohorts were invited to all phases.

We will plan to utilize data from the Cohorts 1 and 2 only (Jan-94 to Aug-95), which were included in all assessments. From the initial 16810 individuals from the 1st Contact, 9410 belonged to Cohorts 1 and 2.

Over the years, sample attrition has led to reductions in the number of participants. For instance, in the TEDS dataset there is data from 3817 at age 8 years, 3412 at age 9 years, 3239 at age 10 years, 3377 at age 12 years and 2776 at age 16 years considering Cohorts 1 and 2.

The actual sample to be used will depend on the extent of missing data, because participants with incomplete data in all of the 5 exposures (outlined in variables) will be excluded from analyses.

Sample size rationale

No response

Stopping rule

No response

Variables

Manipulated variables

No response

No files selected

Measured variables

Exposure data
- Autism Spectrum Disorder (ASD) symptoms: we will plan to utilize the parent-rated CAST ‘social impairments’ (SIs), ‘communication impairments’ (CIs) and the ‘restricted repetitive behaviors and interests’ (RRBIs) subscales at age 8 years as previously done by Ronald and colleagues (Ronald, Happé, Bolton, et al., 2006; Ronald, Happé, Price, Baron-Cohen, & Plomin, 2006).
- Attention-Deficit/Hypearctivity Disorder (ADHD) symptoms: we will plan to use the parent-rated Conners DSM-IV symptom subscale (Inattentive, Hyperactive/Impulsive) scores at age 8 years.
- Learning disorders (LD): we will plan to use the PIAT Reading Comprehension scores and the ‘math tests’ scores at age 10 years.
- Communication disorders (CD): we will plan to use the Children’s Communication Checklist items to derive total scores at age 9 years.
- Intellectual disability (ID): we will plan to use the general cognitive ability ‘g’ composite at age 9 years,

Outcome data
- Depression symptoms and diagnosis in adolescence: we will plan to use the self-reported short Mood and Feelings Questionnaire (sMFQ) total scores at ages 16 years. Diagnosis will be determined by a threshold of > 11 (Thabrew, Stasiak, Bavin, Frampton, & Merry, 2018).
- Depression symptoms in early adulthood: we will plan to use self-reported sMFQ at ages 21 years. Because the sMFQ was abbreviated (8 items) we will not plan to use a diagnosis as determined by a specific threshold (e.g., > 11) in our analyses.

Covariate data
- Emotional dysregulation: we will plan to use the parent-rated Strengths and Difficulties Questionnaire (SDQ) Dysregulation Profile (DP) at age 9 years. The SDQ-DP is composed by SDQ items 2, 8, 12, 13 and 22.
- Peer problems: we will plan to use the SDQ peer problems subscale scores at age 9 years
- Educational attainment: we will plan to use the teacher-rated academic achievement with grades on science, maths and English at 9 years.
- Depression symptoms and diagnosis in childhood: we will plan to use children/parent-reported ‘enhanced’ sMFQ total scores at age 12 years. Diagnosis will be determined by a threshold of > 11 (Thabrew et al., 2018).

No files selected

Indices

No response

No files selected

Analysis Plan

Statistical models

Given this proposal is based on twin data, it would violate assumptions of independence of observations in network estimation. Therefore, analyses will be performed with one twin randomly selected from the pairs of twins available.

We will plan to estimate a total of seven network models ranging from simple to more complex models. In network models, variables (exposure, outcome, and covariate data) are considered ‘nodes’ and ‘edges’ between nodes are conditional dependence relations that can be understood as partial correlations. For network estimation, the R-package ‘mgm’ will be adopted.

A gaussian graphical model will be estimated with only the ND data in childhood. Although measures for different ND have been collected at different ages, those are relatively close from a developmental perspective (ages 8 – 10 years); of note, a similar approach has been previously adopted by previous longitudinal research on ND (Addicoat, Thapar, Riglin, Thapar, & Collishaw, 2020; Eyre et al., 2019). ND will be represented in the network model by total or subscale scores from different instruments. We will plan to utilize scale scores reflecting the sum of several items instead of individual items because, although the scale scores may lead to some loss of information, they improve statistical power because the number of regression coefficients estimated in each model is smaller. Besides, and perhaps more importantly, given that we plan to represent multiple phenotypes in our network, having one unique entry for each phenotype will facilitate the interpretation of our findings.

Next, we will plan to incorporate continuous outcome data (adolescent/adult depression symptom severity) to the ND network. We will plan to estimate separate gaussian graphical models for depression symptom severity in adolescence and early adulthood. For adolescent depression, adolescent -reported data will be preferred. In parallel, we will also incorporate dichotomous outcome data (adolescent depression diagnosis) to the ND network in yet another model. We will plan to estimate mixed graphical models, which enable the use of both continuous and categorical data in the same network. For adolescent depression, adolescent-reported data will be preferred.

Lastly, we will plan to incorporate the covariate data for the two ND network-outcome models in adolescence (ND and depression symptom severity; ND and depression diagnosis) and the ND network-outcome model in early adulthood (ND and depression symptom severity) . For the covariate children depression, children-reported data will be preferred.

Sensitivity analyses
We will plan to use bootstrapping routines as implemented in the package bootnet (Epskamp et al., 2018) to gain information on the precision of parameter estimates from the network models estimated.

We will plan to conduct a sensitivity analysis in which we try to replicate our findings with the other random twin from the same pair.

We will also plan to conduct a sensitivity analysis with parent-reported data of depression in childhood (12 years) and adolescence (16 years).

If the sample size is appropriate, we will plan to estimate separate sets of network models for individuals with and without neurodevelopmental difficulties. Following Eyre et al. (2019) we will plan to categorize individuals as having neurodevelopmental difficulties if they scored in the bottom 5th percentile on at least one of the above-mentioned measures.

Likewise, if the sample size is appropriate, we will plan to estimate networks with only individuals who had complete data (for ND in childhood) without having to perform multiple imputation for ND data.

No files selected

Transformations

Skewed distributions of continuous data will be normalized using the non-paranormal transformation (Liu, Lafferty, & Wasserman, 2009) as recommended for network analysis (Epskamp et al., 2018).

Inference criteria

No response

Data exclusion

No response

Missing data

Although there is no consensus in network analysis, missing data will be imputed through a multiple imputation procedure for those who have complete data on at least one ND to avoid incurring bias due to missing data.

Exploratory analysis

No response

Other

Other

References
Addicoat, A., Thapar, A. K., Riglin, L., Thapar, A., & Collishaw, S. (2020). Adult mood problems in children with neurodevelopmental problems: evidence from a prospective birth cohort followed to age 50. Soc Psychiatry Psychiatr Epidemiol, 55(3), 351-358. doi:10.1007/s00127-019-01727-5
American Psychiatric Association. (2013). Neurodevelopmental Disorders. In Diagnostic and Statistical Manual of Mental Disorders, Fifth Edition. Varlington, VA.
Borsboom, D. (2017). A network theory of mental disorders. World Psychiatry, 16(1), 5-13. doi:10.1002/wps.20375
Coghill, D., & Sonuga-Barke, E. J. (2012). Annual research review: categories versus dimensions in the classification and conceptualisation of child and adolescent mental disorders--implications of recent empirical study. J Child Psychol Psychiatry, 53(5), 469-489. doi:10.1111/j.1469-7610.2011.02511.x
Epskamp, S., Borsboom, D., & Fried, E. I. (2018). Estimating psychological networks and their accuracy: A tutorial paper. Behav Res Methods, 50(1), 195-212. doi:10.3758/s13428-017-0862-1
Eyre, O., Hughes, R. A., Thapar, A. K., Leibenluft, E., Stringaris, A., Davey Smith, G., . . . Thapar, A. (2019). Childhood neurodevelopmental difficulties and risk of adolescent depression: the role of irritability. J Child Psychol Psychiatry, 60(8), 866-874. doi:10.1111/jcpp.13053
Farhat, L. C., Brentani, H., Bastos, V. T., Shephard, E., Mattos, P., Baron-Cohen, S., . . . Polanczyk, G. V. (2021). ADHD and autism symptoms in youth: A network analysis. J Child Psychol Psychiatry, Accepted.
Lai, M. C., Kassee, C., Besney, R., Bonato, S., Hull, L., Mandy, W., . . . Ameis, S. H. (2019). Prevalence of co-occurring mental health diagnoses in the autism population: a systematic review and meta-analysis. Lancet Psychiatry, 6(10), 819-829. doi:10.1016/s2215-0366(19)30289-5
Liu, H., Lafferty, J., & Wasserman, L. (2009). The nonparanormal: semiparametric estimation of high dimensional undirected graphs. The Journal of Machine Learning Research, 10, 2295 - 2328.
Meinzer, M. C., Pettit, J. W., & Viswesvaran, C. (2014). The co-occurrence of attention-deficit/hyperactivity disorder and unipolar depression in children and adolescents: a meta-analytic review. Clin Psychol Rev, 34(8), 595-607. doi:10.1016/j.cpr.2014.10.002
Powell, V., Riglin, L., Hammerton, G., Eyre, O., Martin, J., Anney, R., . . . Rice, F. (2020). What explains the link between childhood ADHD and adolescent depression? Investigating the role of peer relationships and academic attainment. Eur Child Adolesc Psychiatry, 29(11), 1581-1591. doi:10.1007/s00787-019-01463-w
Rice, F., Riglin, L., Thapar, A. K., Heron, J., Anney, R., O'Donovan, M. C., & Thapar, A. (2019). Characterizing Developmental Trajectories and the Role of Neuropsychiatric Genetic Risk Variants in Early-Onset Depression. JAMA Psychiatry, 76(3), 306-313. doi:10.1001/jamapsychiatry.2018.3338
Riglin, L., Leppert, B., Dardani, C., Thapar, A. K., Rice, F., O'Donovan, M. C., . . . Thapar, A. (2020). ADHD and depression: investigating a causal explanation. Psychol Med, 1-8. doi:10.1017/s0033291720000665
Ronald, A., Happé, F., Bolton, P., Butcher, L. M., Price, T. S., Wheelwright, S., . . . Plomin, R. (2006). Genetic heterogeneity between the three components of the autism spectrum: a twin study. J Am Acad Child Adolesc Psychiatry, 45(6), 691-699. doi:10.1097/01.chi.0000215325.13058.9d
Ronald, A., Happé, F., Price, T. S., Baron-Cohen, S., & Plomin, R. (2006). Phenotypic and genetic overlap between autistic traits at the extremes of the general population. J Am Acad Child Adolesc Psychiatry, 45(10), 1206-1214. doi:10.1097/01.chi.0000230165.54117.41
Rouquette, A., Pingault, J. B., Fried, E. I., Orri, M., Falissard, B., Kossakowski, J. J., . . . Borsboom, D. (2018). Emotional and Behavioral Symptom Network Structure in Elementary School Girls and Association With Anxiety Disorders and Depression in Adolescence and Early Adulthood: A Network Analysis. JAMA Psychiatry, 75(11), 1173-1181. doi:10.1001/jamapsychiatry.2018.2119
Thabrew, H., Stasiak, K., Bavin, L. M., Frampton, C., & Merry, S. (2018). Validation of the Mood and Feelings Questionnaire (MFQ) and Short Mood and Feelings Questionnaire (SMFQ) in New Zealand help-seeking adolescents. Int J Methods Psychiatr Res, 27(3), e1610. doi:10.1002/mpr.1610
Thapar, A., Cooper, M., & Rutter, M. (2017). Neurodevelopmental disorders. Lancet Psychiatry, 4(4), 339-346. doi:10.1016/s2215-0366(16)30376-5

Contributors
Luis Carlos Farhat and Elizabeth Shephard

Description

Show more

Registration type
OSF Preregistration

Date registered
June 30, 2021

Date created
June 30, 2021

Associated project
osf.io/p7wmk

Internet Archive link
https://archive.org/details/osf-registrations-qp7zm-v1

Category
Project

Registration DOI
https://doi.org/10.17605/OSF.IO/QP7ZM

Subjects
Mental and Social Health
Medicine and Health Sciences
Psychiatry and Psychology

License
CC0 1.0 Universal

Citation
osf.io/qp7zm

Copyright © 2011-2023 Center for Open Science | Terms of Use | Privacy Policy | Status | API
TOP Guidelines | Reproducibility Project: Psychology | Reproducibility Project: Cancer Biology
This website relies on cookies to help provide a better user experience. By clicking Accept or continuing to use the site, you agree. For more information, see our Privacy Policy and information on cookie use.
Accept

Supplement B. Cohort descriptions.

The Twins Early Development Study (TEDS) is a twin cohort that recruited participants from all live twin births in England or Wales between January 1994 and December 1996. Birth records were used to identify 16,810 families, of which 13,945 consented and provided data to the study in at least the first of the 13 core waves of data collection over the 21 years of follow-up. The TEDS sample was representative of the UK population at first contact and remains broadly representative despite attrition. Full details of the study design, sample, and measures have been provided elsewhere (10). Additional details of each study variable can be found in the TEDS online data dictionary (see https://www.teds.ac.uk/datadictionary/home.htm). TEDS is divided into cohorts according to twin birth dates. Not all cohorts, or families within cohorts, were invited to participate in every wave due to budget constraints and to avoid overburdening families. As many as 5,554 families (11,108 individuals) were invited to participate in all waves of data collection of interest to the current analyses.

The Avon Longitudinal Study of Parents And Children (ALSPAC) is a birth cohort that recruited participants from all pregnant women living in Avon, UK, with expected dates of delivery between 1st April 1991 to 31st December 1992. Additional post-natal recruitment efforts were made to include eligible cases who had failed to join the study originally (11-13). In total, there were 15,454 pregnancies and 15,589 fetuses, resulting in 14,901 children alive at 1 year of age. Ethical approval for the study was obtained from the ALSPAC Ethics and Law Committee and the Local Research Ethics Committees. Informed consent for the use of data collected via questionnaires and clinics was obtained from participants following the recommendations of the ALSPAC Ethics and Law Committee at the time. Please note that the study website contains details of all the data that is available through a fully searchable data dictionary and variable search tool (http://www.bristol.ac.uk/alspac/researchers/our-data/).

Supplement C. Additional details of variables, measurements and scoring rules.

Most variables are questionnaire-based and were collected through web-based forms or pencil-and-paper booklets, while others were collected as web tests or in-person assessments. Measures were completed by parents, teachers, or the individuals themselves. Most measures are validated, but to reduce burden on participants, only a subset of items of the instruments measuring communication ability in both cohorts and depression in TEDS at age 21 years were administered. Additionally, measures of cognitive and learning abilities involved subsets and/or adaptations of validated tests.

Autistic symptoms

The Childhood Autism Spectrum Test (CAST, formerly known as Childhood Asperger Syndrome Test) (14) is a parent-rated questionnaire which was designed to screen school-aged children for the diagnosis of autism. The CAST was composed based on descriptions of the International Classification of Diseases, 10th edition (ICD-10)/Diagnostic Statistical Manual of Mental Disorders, fourth edition (DSM-IV) core features of autism, and therefore it is consistent with the classical triad of SIs, CIs and RRBIs. The CAST has good psychometric properties (15, 16). It is composed of 37 items of which only 31 were scored dichotomously (yes/no) as the remaining 6 items are control questions on general development. Total autistic symptom scores were computed by summing the 31 items. Items 1, 2, 3, 6, 8, 9, 10, 12, 13, 14, 18, 19, 20, 22, 26, and 29 were reversed scored. At least 16 items were required to be non-missing to compute prorated scores. Higher scores indicate more autistic symptoms.

The Social and Communication Disorder Checklist (SCDC) (17) is a parent-rated questionnaire aimed at assessing social cognition over the past 6 months. The SCDC has sound psychometric properties to detect autistic symptoms in the general population (17), although it might lack specificity to separate autism from other psychiatric conditions with social communication impairments (18). The SCDC is composed of 12 items which were rated 0 ('Not at all true'), 1 ('Sometimes true) and 2 ('Often true'). Total social cognition scores were computed by summing the 12 items. At least 6 of the items were required to be non-missing to compute prorated scores. Higher scores indicate more autistic symptoms.

Attention-deficit/hyperactivity disorder (ADHD) symptoms

The Conners parent rating scale-revised (CPRS-R) (19) contains 18 items which query about symptoms of DSM-IV defined ADHD (DSM-IV symptom subscale) over the past month. The CPRS-R has sound psychometric properties in school-aged children (19). Items were rated 0 ('not true at all'), 1 ('just a little true'), 2 ('pretty much true') or 3 ('very much true'). Continuous scores for each subscale (hyperactivity/impulsivity, inattention) were computed by summing the 9 items of each subscale separately. For both subscales, at least 5 of the items were required to be non-missing to compute prorated scores. Higher scores indicate more ADHD symptoms.

The Development and Well-Being Assessment (DAWBA) (20) is a structured interview (yes/no) for children aged 4-16 years old administered by lay interviewers which integrates information from multiple informants (e.g., caretakers, teachers and self-reports for children aged 11 years or older). In Britain, DAWBA has demonstrated good reliability and discriminative ability for the diagnosis of multiple psychiatric conditions including ADHD (20). In ALSPAC, the DAWBA was used as a parent-reported questionnaire only. "*Section K: Attention and activity*" queries about ADHD symptoms over the past 6 months with questions closely related to

DSM-IV operational criteria. Items were rated 0 (‘No’), 1 (‘A little more than others’) and 2 (‘A lot more than others’). Continuous scores for each subscale (hyperactivity/impulsivity and inattention) were computed by summing the 9 items of each subscale separately. For both subscales, at least 5 of the items were required to be non-missing to compute prorated scores. Higher scores indicate more ADHD symptoms.

General cognitive ability

The general cognitive ability (*g*) score was created as the average of standardized scores on two verbal and non-verbal tests. The two verbal tests were adapted from the Wechsler Intelligence Scale for Children-III (WISC-III) (21). One of the verbal tests involved general knowledge questions (e.g., ‘on what continent is Brazil?’) to evaluate the individual’s ability to acquire, retain and retrieve information. The other verbal test involved vocabulary questions (e.g., ‘what does rivalry mean’) to measure knowledge and verbal concept formation. The two non-verbal tests were adapted from the Cognitive Abilities Test 3 (CAT3) (22). One of the tests asked children to identify the shape out of five which would continue a series to measure inductive reasoning and visualization. The other test asked children to identify the one shape out of five that would be related to another shape in a similar way as a given example (e.g., a rectangle and a square relate to each other like an oval and what other shape?) to measure inductive and deductive reasoning. Higher scores indicate higher general cognitive ability.

The full-scale intelligence quotient (IQ) score was derived from ten shortened subtests adapted from the WISC-III, five each for verbal (vocabulary, similarities, arithmetic, information, comprehension) and performance/non-verbal (object assembly, coding, block design, picture arrangement, picture completion) subtests. Children were required to have at least 8 subtests (with more than 4 for each verbal/performance domain) to compute prorated scores. Higher scores indicate higher general cognitive ability.

Communication ability

The Children’s Communication Checklist (CCC) (23) is a parent-rated questionnaire aimed at measuring communication impairments in children. The CCC is composed of 70 items divided in nine subscales (subscales A to I), five of which (subscales C to G) are summed to compose a measure of pragmatic aspects of communication. In the original description of the instrument, items are negative and rated ‘does not apply’ (0), ‘applies somewhat’ (-1), and ‘definitely applies’ (-2). Some of the items are positive, please refer to the article by Bishop (23) for a comprehensive description. Higher scores indicate better communication ability (23). The CCC has sound psychometric properties in school-aged children.(23).

To avoid overburdening families, a shortened version of the scale with 53 items (subscales A to G) was used in ALSPAC. Items were positively scored on a 3-point Likert scale and some items were reverse scored. Please refer to the ALSPAC dictionary for additional details about scoring rules of each item. The sum of subscales A and B correspond to the speech & syntax score, whereas the sum of subscales C to G correspond to the pragmatic score – as defined above. A minimum of 6 (subscale A), 4 (subscale D to G), 3 (subscale C) and 2 (subscale B) items were required to be non-missing to compute prorated scores.

In TEDS, an even shorter version with only 12 items (5 items from subscale A; 3 items from subscale B; and 4 items from subscale D) of the original 70-item instrument was applied. Items were rated considering the same rules adopted for ALSPAC. We computed subscale scores for TEDS by summing the 8 items from subscales A and B (speech & syntax) and the 4 items from subscale D (pragmatic) separately. We required at least 4 and 2 items to be non-missing to compute prorated speech & syntax and pragmatic scores, respectively.

Learning ability

The learning ability score from TEDS was based on an adaptation of the reading comprehension subtest from the Peabody Individual Achievement Test (PIAT), which assesses literal comprehension of sentences (24). In each item, the child was asked to read a sentence and then choose the picture out of four that best illustrated the sentence. There were 89 total items of which 82 were scored. Vocabulary level of sentences increased through the upper grade material. Each item (including text, pictures, and correct responses) is described in the TEDS data dictionary (see https://www.teds.ac.uk/datadictionary/studies/webtests/10yr_PIAT_test.htm). Higher scores indicate higher learning ability.

The learning ability score from ALSPAC comprised the basic reading subtest of the Wechsler Objective Reading Dimensions (WORD) (25). There were three tasks involved in this test. First, the child was shown a picture and had to choose the word out of four that had the same beginning or ending sound as the picture. Second, the child was asked to select the word out of four that matched the picture. Lastly, the child was asked to read aloud a list of 48 unconnected words that increased in difficulty. The number of correct items composed the total score. Higher scores indicate higher learning ability.

Peer relationships

The strengths and difficulties questionnaire peer relationships problem subscale (SDQ-PP) is a parent-rated questionnaire which evaluates how well their children get on with other children over the past 6 months or the present school year. There are 5 items which were rated as ‘not true’ (0), ‘somewhat true’ (1), and ‘certainly true’ (2). The SDQ has sound psychometric properties in school-aged children (26). Higher scores indicate the child has more problems in their relations with other children. In both TEDS and ALSPAC, at least 3 of the items were required to be non-missing to compute prorated scores.

Academic competence

The ‘national curriculum’ (NC) is a set of subjects and standards used by primary and secondary school in the UK to ensure children learn the same subjects in the country. At the end of key stages (KS), NC assessment of each pupil is made by direct testing and teacher assessment. The teacher assessment score for a particular child ultimately determines the final score that is submitted to the qualifications and curriculum authority. The KS2 assessment correspond to the period covering school years 3-6, when children are aged ~7-11 years old. In TEDS, teachers were asked to check one of five boxes to indicate the child’s NC teacher assessment score (27). In ALSPAC, the KS2 score was obtained through linkage with the national pupil database (NPD). For details for details on scoring, please refer to https://education.infotap.uk/schools/performance/archive/schools_10/points.pdf.

Co-occurring emotional dysregulation

The strengths and difficulties questionnaire dysregulation profile subscale (SDQ-DP) (28) was created to measure a broad dysregulation phenotype in a similar manner to the Children’s Behavior Checklist (CBCL) (29). The SDQ-DP comprises 5 items which reflect affective and behavioural dysregulation (‘often unhappy, down hearted or tearful’; ‘many worries, often seems worried’; ‘often fights with other children or bullies them’; ‘steals from home, school or elsewhere’; ‘restless, overactive, cannot stay still for long’) over the past 6 months or school year. Items were rated ‘not true’ (0), ‘somewhat true’ (1), and ‘certainly true’ (2). The SDQ-DP has

sound psychometric properties (28, 30). In both TEDS and ALSPAC, we required at least 3 items to be non-missing to compute prorated scores.

Depressive symptoms

The short mood and feelings questionnaire (sMFQ) is a questionnaire derived from the 33-item MFQ (31) which evaluates core depressive symptoms over the past two weeks. The sMFQ has sound psychometric properties in both children/adolescents (32) and young adults (33). The sMFQ has 13 items which are rated as 'not true' (0), 'sometimes true' (1) and 'true' (2). Total scores were computed by summing the 13 items, but in TEDS for adults (21 years) total scores were generated by summing the 8 items which were administered. In TEDS, prorated scores were computed if at least 7 items were not missing for children (12 years) and adolescents (16 years); for adults (21 years), if at least 4 items were not missing. In ALSPAC, sMFQ scores were collected through in-person assessments and complete data were available.

Supplement D. Exclusions of individuals from the analyses

Of the 11,108 individuals from TEDS, we randomly selected one twin from each pair and then excluded 1,147 individuals because they fulfilled criteria for perinatal outliers, had a medical condition which would likely affect their participation in TEDS, or which was known to be associated with mental impairments (see https://www.teds.ac.uk/datadictionary/exclusions.htm), or had all neurodevelopmental trait data missing, i.e., did not provide data on any of the neurodevelopmental traits of interest to the study.

Of the 14,901 individuals from ALSPAC, we excluded 4,550 individuals because they had all neurodevelopmental trait data missing.

Supplement E. Missing data per variable

Table S1. Number and proportion of missing data of each variable in the Twins Early Development Study (TEDS) and the Avon Longitudinal Study of Parents And Children (ALSPAC)

	TEDS (N = 4,407)	ALSPAC (N = 10,351)
	n (%)	n (%)
A	707 (16.04)	2,529 (24.43)
HY	708 (16.06)	2,294 (22.16)
IN	710 (16.11)	2,304 (22.26)
GEN COG	1,201 (27.25)	3,104 (29.99)
LD-R	1,464 (33.22)	2,402 (24.09)
C-P	1,097 (24.89)	2,494 (22.57)
C-SS	1,097 (24.89)	2,336 (23.12)
DP	1,079 (24.48)	2,393 (23.06)
PP	1,078 (24.46)	2,387 (23.06)
EA	1,711(38.82)	8,828 (85.29)
C-DEP	1,374 (31.18)	3,257 (31.47)
ADO-DEP	2,071 (46.99)	5,566 (53.77)
ADU-DEP	2,130 (48.33)	7,203 (69.59)

Abbreviations: A = Autistic symptoms; ADO-DEP = Adolescent depressive symptoms; ADU-DEP = Adult depressive symptoms; C-DEP = Childhood depressive symptoms; C-P = Pragmatic sub-domain of communication ability; C-SS = Speech & syntax sub-domain of communication ability; DP = Dysregulation Profile; EA = Educational Attainment; GEN COG = General cognitive ability; HY = Hyperactivity/impulsivity sub-domain of ADHD symptoms; IN = Inattention sub-domain of ADHD symptoms; LD-R = Learning ability; PP = Peer Problems.

Supplement F. Bayes Factor values for every pair of variables.

Table S2. Bayes Factor (BF_{10}) in support of $\mathcal{H}_1: \rho \neq 0$ against $\mathcal{H}_0: \rho = 0$ and vice-versa (BF_{01}) for each pair of variables in the Twins Early Development Study (TEDS) and the Avon Longitudinal Study of Parents And Children (ALSPAC)

	TEDS		ALSPAC	
	BF_{10}	BF_{01}	BF_{10}	BF_{01}
A & HY	Inf	0	Inf	0
A & IN	Inf	0	Inf	0
A & C-P	Inf	0	Inf	0
A & C-SS	Inf	0	0.64	1.57
A & GEN COG	0.23	4.39	0.08	11.78
A & LD-R	0.11	8.79	0.27	3.64
HY & IN	Inf	0	Inf	0
HY & C-P	0.67	1.49	Inf	0
HY & C-SS	0.19	5.22	Inf	0
HY & GEN COG	0.36	2.74	0.55	1.82
HY & LD-R	0.34	2.96	0.55	1.82
IN & C-P	Inf	0	Inf	0
IN & C-SS	0.51	1.96	Inf	0
IN & GEN COG	Inf	0	1052.25	0.001
IN & LD-R	0.16	6.37	Inf	0
C-P & C-SS	Inf	0	Inf	0
C-P & GEN COG	0.08	12.66	Inf	0
C-P & LD-R	48.05	0.021	318.15	0.003
C-SS & GEN COG	1034.38	0.001	108.61	0.01
C-SS & LD-R	8.04	0.12	Inf	0
GEN COG & LD-R	Inf	0	Inf	0
A & DP	1.8	0.56	Inf	0
A & PP	Inf	0	Inf	0
A & EA	0.09	10.72	0.12	8.60
A & C-DEP	0.1	10.28	0.32	3.09
A & ADO-DEP	0.18	5.68	0.14	7.32
A & ADU-DEP	0.19	5.20	0.08	12.01
HY & DP	Inf	0	Inf	0
HY & PP	0.11	8.97	1.13	0.89
HY & EA	1.96	0.51	1.87	0.53
HY & C-DEP	29.95	0.03	0.13	7.50
HY & ADO-DEP	0.46	2.20	0.09	11.46
HY & ADU-DEP	0.13	7.47	0.09	11.30
IN & DP	Inf	0	0.13	7.61
IN & PP	0.65	1.54	Inf	0
IN & EA	Inf	0	0.3	3.30
IN & C-DEP	0.23	4.35	0.21	4.79
IN & ADO-DEP	0.18	5.48	0.1	10.38
IN & ADU-DEP	0.16	6.10	0.1	10.41
GEN COG & DP	0.23	4.35	0.09	11.14
GEN COG & PP	0.12	8.19	0.08	12.11
GEN COG & EA	Inf	0	Inf	0
GEN COG & C-DEP	1.65	0.61	2.13	0.47
GEN COG & ADO-DEP	0.12	8.61	0.38	2.61
GEN COG & ADU-DEP	0.13	7.42	0.19	5.23
LD-R & DP	0.36	2.79	0.19	5.16
LD-R & PP	0.09	11.04	0.87	1.16
LD-R & EA	Inf	0	Inf	0
LD-R & C-DEP	0.33	3	1.42	0.7
LD-R & ADO-DEP	0.11	8.73	0.72	1.38
LD-R & ADU-DEP	0.13	7.91	0.22	4.65

C-P & DP	0.58	1.73	Inf	0
C-P & PP	0.15	6.64	Inf	0
C-P & EA	0.69	1.45	6.66	0.15
C-P & C-DEP	0.09	10.78	0.12	8.61
C-P & ADO-DEP	10.34	0.10	0.12	8.15
C-P & ADU-DEP	0.34	2.98	0.13	7.55
C-SS & DP	1.66	0.60	0.35	2.88
C-SS & PP	56.11	0.02	Inf	0
C-SS & EA	13.63	0.07	96.4	0.01
C-SS & C-DEP	0.17	5.95	0.08	13.36
C-SS & ADO-DEP	0.14	7.25	0.11	8.83
C-SS & ADU-DEP	0.12	8.05	0.78	1.28
DP & PP	Inf	0	Inf	0
DP & EA	0.56	1.8	0.16	6.09
DP & C-DEP	Inf	0	8.48	0.12
DP & ADO-DEP	0.34	2.92	58.02	0.02
DP & ADU-DEP	0.17	5.88	0.08	12.29
PP & EA	0.17	5.88	0.13	7.86
PP & C-DEP	72.47	0.01	Inf	0
PP & ADO-DEP	3.88	0.26	0.06	15.62
PP & ADU-DEP	0.68	1.48	47.24	0.02
EA & C-DEP	0.45	2.22	4.47	0.22
EA & ADO-DEP	2.19	0.46	0.18	5.73
EA & ADU-DEP	3.08	0.32	0.18	5.58
C-DEP & ADO-DEP	Inf	0	Inf	0
C-DEP & ADU-DEP	158.11	0.01	Inf	0
ADO-DEP & ADU-DEP	Inf	0	Inf	0

Abbreviations: A = Autistic symptoms; ADO-DEP = Adolescent depressive symptoms; ADU-DEP = Adult depressive symptoms; C-DEP = Childhood depressive symptoms; C-P = Pragmatic sub-domain of communication ability; C-SS = Speech & syntax sub-domain of communication ability; DP = Dysregulation Profile; EA = Educational Attainment; GEN COG = General cognitive ability; HY = Hyperactivity/impulsivity sub-domain of ADHD symptoms; IN = Inattention sub-domain of ADHD symptoms; LD-R = Learning ability; PP = Peer Problems.

Supplement G. Zero-order correlations

Table S3. Pearson's correlation for each pair of variables in the Twins Early Development Study (TEDS) and the Avon Longitudinal Study of Parents And Children (ALSPAC)

	TEDS		ALSPAC	
	r (95% CI)	p	r (95% CI)	p
**A & C-DEP**	**0.17 (0.14, 0.20)**	**< $2.2x10^{-16}$**	**0.11 (0.09, 0.13)**	**< $2.2x10^{-16}$**
**A & ADO-DEP**	**0.09 (0.06, 0.11)**	**$1x10^{-8}$**	**0.07 (0.05, 0.09)**	**$8.7x10^{-12}$**
**A & ADU-DEP**	**0.05 (0.02, 0.08)**	**$3.6x10^{-4}$**	**0.07 (0.05, 0.09)**	**$1.1x10^{-12}$**
HY & C-DEP	0.24 (0.21, 0.26)	< $2.2x10^{-16}$	0.07 (0.05, 0.09)	$1.7x10^{-13}$
HY & ADO-DEP	0.08 (0.05, 0.11)	$2.3x10^{-8}$	0.04 (0.02, 0.06)	$2.3x10^{-5}$
**HY & ADU-DEP**	**0.07 (0.04, 0.09)**	**$1.1x10^{-5}$**	**0.08 (0.06, 0.09)**	**$8.5x10^{-15}$**
**IN & C-DEP**	**0.23 (0.20, 0.26)**	**< $2.2x10^{-16}$**	**0.08 (0.06, 0.10)**	**< $2.2x10^{-16}$**
**IN & ADO-DEP**	**0.13 (0.1, 0.16)**	**< $2.2x10^{-16}$**	**0.05 (0.03, 0.07)**	**$1.4x10^{-7}$**
**IN & ADU-DEP**	**0.11 (0.08, 0.14)**	**$1.1x10^{-12}$**	**0.08 (0.06, 0.09)**	**$9.3x10^{-15}$**
GEN COG & C-DEP	-0.17 (-0.19, -0.14)	< $2.2x10^{-16}$	-0.007 (-0.03, 0.01)	0.46
GEN COG & ADO-DEP	-0.04 (-0.07, -0.01)	$4.4x10^{-4}$	-0.02 (-0.04, -0.0040	0.01
GEN COG & ADU-DEP	-0.06 (-0.09, -0.04)	$1.3x10^{-5}$	-0.008 (-0.03, 0.01)	0.40
LD-R & C-DEP	-0.19 (-0.22, -0.17)	< $2.2x10^{-16}$	0.02 (0.01, 0.04)	$8.2x10^{-3}$
LD-R & ADO-DEP	-0.07 (-0.10, -0.04)	$1.8x10^{-6}$	0.01 (-0.005, 0.03)	0.17
LD-R & ADU-DEP	-0.07 (-0.10, -0.04)	$1.4x10^{-6}$	0.002 (-0.02, 0.02)	0.80
**C-SS & C-DEP**	**-0.10 (-0.13, -0.07)**	**$3.3x10^{-11}$**	**-0.05 (-0.07, -0.03)**	**$1x10^{-7}$**
C-SS & ADO-DEP	-0.08 (-0.11, -0.05)	$1x10^{-7}$	-0.02 (-0.04, 0)	0.06
C-SS & ADU-DEP	-0.03 (-0.06, -0.003)	0.03	-0.02 (-0.04, -0.001)	0.03
**C-P & C-DEP**	**-0.13 (-0.16, -0.10)**	**< $2.2x10^{-16}$**	**-0.08 (-0.10, -0.06)**	**< $2.2x10^{-16}$**
C-P & ADO-DEP	-0.12 (-0.15, -0.09)	< $1.3x10^{-15}$	-0.09 (-0.11, -0.08)	< $2.2x10^{-16}$
C-P & ADU-DEP	-0.02 (-0.05, 0.01)	0.21	-0.10 (-0.11, -0.08)	< $2.2x10^{-16}$

Values represent Pearson's r with 95% confidence intervals.
Bold indicates associations for which zero-order correlations were statistically significant at a Bonferroni-adjusted $p < 2.38*10^{-3}$ in both cohorts and for which there was sufficient evidence of conditional independence based on GGM analyses.
Grey shading indicates associations for which zero-order correlations were statistically significant at a Bonferroni-adjusted $p < 2.38*10^{-3}$ in both cohorts and for which evidence from GGMs was inconclusive (i.e., incongruent across cohorts or ambiguous in at least one cohort).
Abbreviations: A = Autistic symptoms; ADO-DEP = Adolescent depressive symptoms; ADU-DEP = Adult depressive symptoms; C-DEP = Childhood depressive symptoms; C-P = Pragmatic sub-domain of communication ability; C-SS = Speech & syntax sub-domain of communication ability; GEN COG = General cognitive ability; HY = Hyperactivity/impulsivity sub-domain of ADHD symptoms; IN = Inattention sub-domain of ADHD symptoms; LD-R = Learning ability.

Supplement H. Predictability values for each variable.

Table S4. Bayesian R^2 for each pair of variables in the Twins Early Development Study (TEDS) and the Avon Longitudinal Study of Parents And Children (ALSPAC)

	TEDS	ALSPAC
	R^2 (95% CrI)	R^2 (95% CrI)
A	0.36 (0.34, 0.38)	0.43 (0.41, 0.44)
HY	0.51 (0.50, 0.53)	0.58 (0.57, 0.60)
IN	0.50 (0.48, 0.52)	0.54 (0.52, 0.55)
GEN COG	0.30 (0.28, 0.32)	0.46 (0.45, 0.48)
LD-R	0.30 (0.28, 0.32)	0.39 (0.38, 0.40)
C-P	0.30 (0.27, 0.31)	0.41 (0.40, 0.42)
C-SS	0.27 (0.25, 0.29)	0.14 (0.13, 0.15)
DP	0.38 (0.36, 0.40)	0.30 (0.29, 0.32)
PP	0.30 (0.28, 0.32)	0.26 (0.24, 0.27)
EA	0.34 (0.32, 0.36)	0.48 (0.47, 0.49)
C-DEP	0.18 (0.16, 0.20)	0.17 (0.16, 0.18)
ADO-DEP	0.21 (0.20, 0.23)	0.22 (0.21, 0.23)
ADU-DEP	0.17 (0.15, 0.19)	0.20 (0.18, 0.21)

Values represent posterior means (95% credible intervals).
Abbreviations: A = Autistic symptoms; ADO-DEP = Adolescent depressive symptoms; ADU-DEP = Adult depressive symptoms; C-DEP = Childhood depressive symptoms; C-P = Pragmatic sub-domain of communication ability; C-SS = Speech & syntax sub-domain of communication ability; DP = Dysregulation Profile; EA = Educational Attainment; GEN COG = General cognitive ability; HY = Hyperactivity/impulsivity sub-domain of ADHD symptoms; IN = Inattention sub-domain of ADHD symptoms; LD-R = Learning ability; PP = Peer Problems.

Supplement I. Standardized regression coefficients

Table S5. Standardized regression coefficients for each pair of variables in the Twins Early Development Study (TEDS) and the Avon Longitudinal Study of Parents And Children (ALSPAC)

**Twins Early Development Study (TEDS)**						
	DP	PP	EA	C-DEP	ADO-DEP	ADU-DEP
	β (95% CrI)	β (95% CrI)	β (95% CrI)	β (95% CrI)	β (95% CrI)	β (95% CrI)
A		0.27 (0.24, 0.31)	0	0	0	0
HY	0.28 (0.24, 0.32)					0
IN				0	0	0
GEN COG	0	0	0.24 (0.21, 0.28)			0
LD-R			0.27 (0.23, 0.30)			0
C-P				0		
C-SS		-0.06 (-0.10, -0.03)	0.07 (0.02, 0.11)	0	0	
DP	NA	0.29 (0.26, 0.33)		0.10 (0.06, 0.14)		0
PP	0.26 (0.23, 0.29)	NA	0	0.08 (0.04, 0.12)		
EA		0	NA			
**Avon Longitudinal Study of Parents & Children (ALSPAC)**						
	DP	PP	EA	C-DEP	ADO-DEP	ADU-DEP
	β (95% CrI)	β (95% CrI)	β (95% CrI)	β (95% CrI)	β (95% CrI)	β (95% CrI)
A		0.09 (0.06, 0.12)	0	0	0	0
HY	0.21 (0.18, 0.24)					0
IN				0	0	0
GEN COG	0	0	0.40 (0.34, 0.45)			0
LD-R			0.27 (0.21, 0.33)			0
C-P				0		
C-SS		-0.06 (-0.08, -0.03)	0.09 (0.02, 0.14)	0	0	
DP	NA	0.25 (0.23, 0.27)		0.05 (0.02, 0.08)		0
PP	0.23 (0.21, 0.25)	NA	0	0.08 (0.05, 0.12)		
EA		0	NA			

Values represent posterior means (95% credible intervals).
Regression coefficients considering domains in columns as the dependent variables (Y) and domains in rows as the independent variables (X). The index of mediation is calculated by multiplying the regression coefficients as previously described (34).
Grey shading indicates associations for which findings across TEDS and ALSPAC were either ambiguous in at least one cohort or discordant across cohorts.
Abbreviations: A = Autistic symptoms; ADO-DEP = Adolescent depressive symptoms; ADU-DEP = Adult depressive symptoms; C-DEP = Childhood depressive symptoms; C-P = Pragmatic sub-domain of communication ability; C-SS = Speech & syntax sub-domain of communication ability; DP = Dysregulation Profile; EA = Educational Attainment; GEN COG = General cognitive ability; HY = Hyperactivity/impulsivity sub-domain of ADHD symptoms; IN = Inattention sub-domain of ADHD symptoms; LD-R = Learning ability; NA = Not applicable; PP = Peer Problems.

Supplement J. Sensitivity analyses

1. Decreasing the prior scale (SD = 0.1)

Summary of changes from the main analyses

Neurodevelopmental traits.

In TEDS, the association between autistic symptoms and general cognitive ability was classified as ambiguous. In ALSPAC, the association between autistic symptoms and learning ability was classified as ambiguous.

Neurodevelopmental traits, social-environmental stressors, and co-occurring emotional dysregulation.

In TEDS, the general cognitive ability and emotional dysregulation as well as inattention symptoms and childhood depressive symptoms were classified as ambiguous. In ALSPAC, childhood depressive symptoms and autistic, inattention symptoms were classified as ambiguous. Likewise, learning ability and adult depressive symptoms was classified as ambiguous.

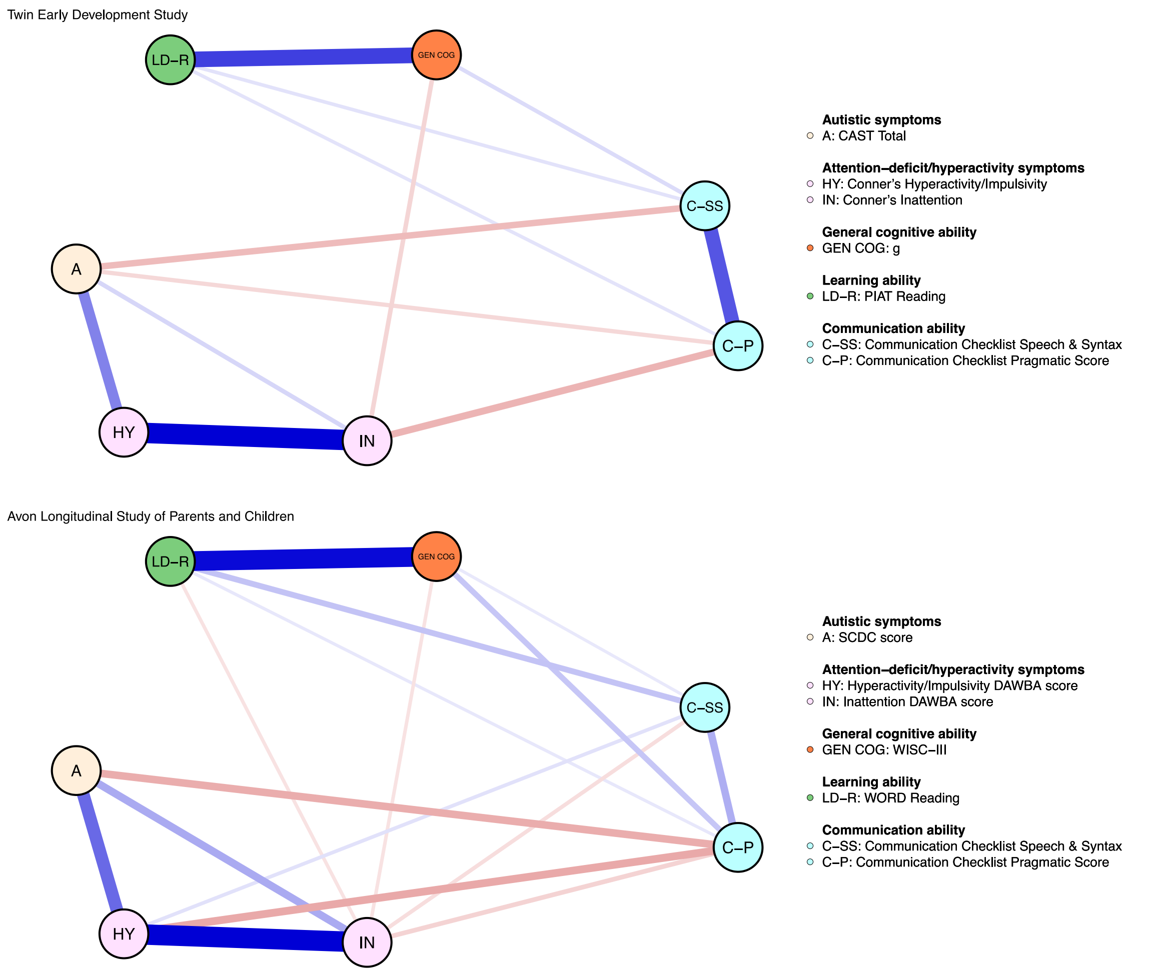

Figure S1. Network plots for neurodevelopmental traits in childhood (7-10 years old) in the Twins Early Development Study (top) and the Avon Longitudinal Study of Parents And Children (bottom) after decreasing the prior scale. Variables are represented as nodes (circles) and are colored according to their domain. Edges between two nodes represent partial correlations between two variables. The width of edges is proportional to the strength of the partial correlation. Positive and negative partial correlations were colored in blue and red, respectively.

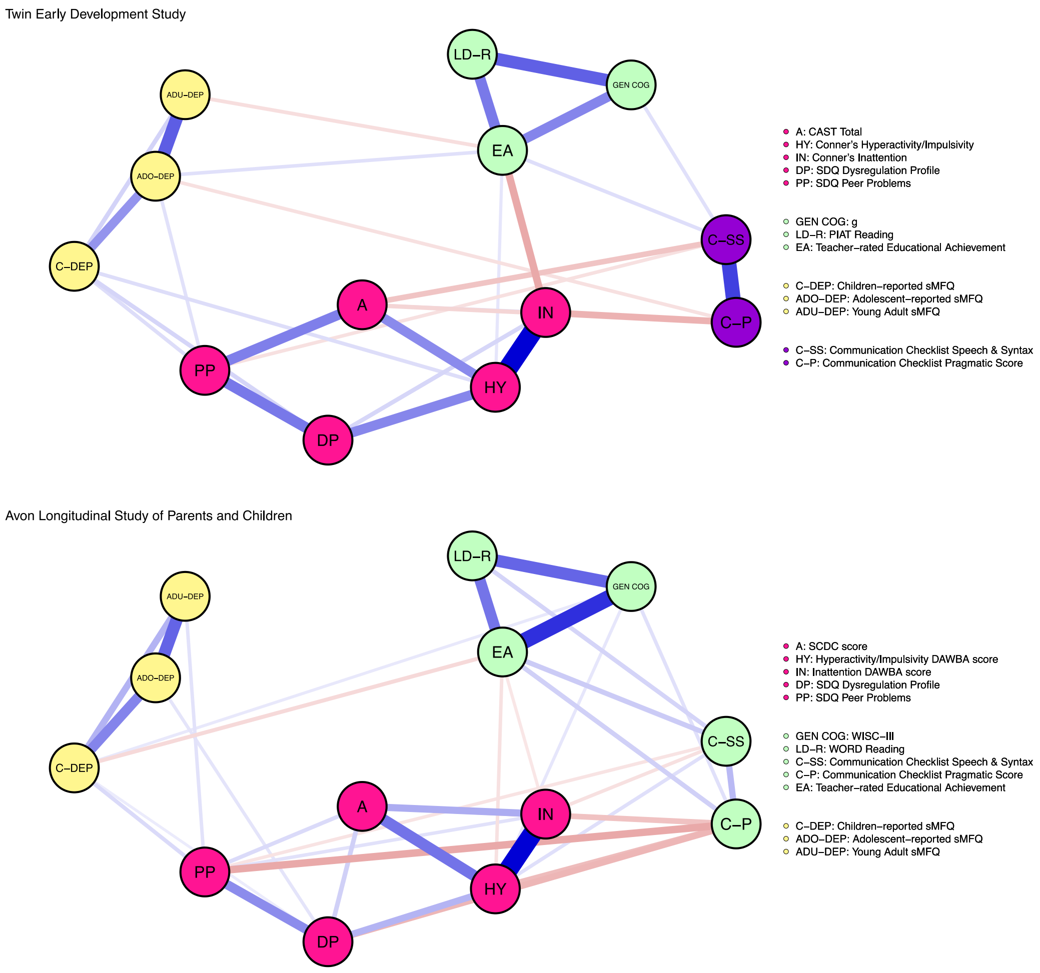

Figure S2. Network plots for neurodevelopmental traits, social-environmental stressors, and co-occurring emotional dysregulation in childhood (7-11 years old) and depressive symptoms over development in childhood (12 years), adolescence (16 years) and adulthood (21 years) in the Twins Early Development Study (top) and the Avon Longitudinal Study of Parents And Children (bottom) after decreasing the prior scale. Variables are represented as nodes (circles) and are colored according to their community as identified by the spinglass algorithm. Edges between two nodes represent partial correlations between two variables. The width of edges is proportional to the strength of the partial correlation. Positive and negative partial correlations were colored in blue and red, respectively.

Table S4. Conditional associations between neurodevelopmental traits, social-environmental stressors, co-occurring emotional dysregulation, and depressive symptoms, as well as their relative magnitude, for the Twins Early Development Study (lower triangle) and the Avon Longitudinal Study of Parents & Children (upper triangle) after decreasing the prior scale.

A	0.32 (0.30, 0.34)	0.19 (0.16, 0.21)	0	Ambiguous‡	Ambiguous†	-0.18 (-0.20, -0.16)	0.10 (0.07, 0.13)	0.09 (0.06, 0.10)	0	Ambiguous‡	0	0
0.29 (0.26, 0.32)	*HY*	0.51 (0.49, 0.53)	Ambiguous†	Ambiguous†	0.05 (0.04, 0.09)	-0.18 (-0.21, -0.16)	0.17 (0.14, 0.18)	Ambiguous†	-0.03† (-0.11, -0.01)	0	0	0
0.10 (0.06, 0.13)	0.54 (0.52, 0.57)	*IN*	-0.08 (-0.09, -0.04)	-0.05 (-0.09, -0.04)	-0.06 (-0.1, -0.05)	-0.10 (-0.12, -0.07)	0	0.07 (0.04, 0.09)	Ambiguous‡	Ambiguous‡	0	0
Ambiguous‡	Ambiguous†	-0.14 (-0.14, -0.06)	*GEN COG*	0.50 (0.47, 0.52)	0.05 (0.03, 0.08)	0.11 (0.10, 0.15)	0	0	0.44 (0.36, 0.46)	0.02† (0.01, 0.09)	Ambiguous†	0
0	Ambiguous†	0	0.44 (0.40, 0.46)	*LD-R*	0.13 (0.11, 0.16)	0.07 (0.03, 0.08)	0	Ambiguous†	0.26 (0.23, 0.35)	Ambiguous†	Ambiguous†	Ambiguous‡
-0.18 (-0.20, -0.12)	0	Ambiguous†	0.07 (0.05, 0.12)	0.09 (0.03, 0.12)	*C-SS*	0.17 (0.15, 0.20)	Ambiguous†	-0.06 (-0.08, -0.04)	0.14 (0.03, 0.18)	0	0	Ambiguous†
-0.10 (-0.13, -0.06)	Ambiguous†	-0.18 (-0.21, -0.14)	0	0.07 (0.03, 0.11)	0.39 (0.35, 0.42)	*C-P*	-0.15 (-0.18, -0.13)	-0.19 (-0.20, -0.16)	0.11 (0.03, 0.07)	0	0	0
Ambiguous†	0.26 (0.21, 0.28)	0.08 (0.05, 0.12)	Ambiguous‡	Ambiguous†	Ambiguous†	Ambiguous†	*DP*	0.24 (0.22, 0.26)	0	0.06 (0.02, 0.07)	0.04 (0.03, 0.09)	0
0.26 (0.23, 0.29)	0	Ambiguous†	0	0	-0.06 (-0.10, -0.03)	0	0.26 (0.24, 0.31)	*PP*	0	0.08 (0.05, 0.11)	0	0.09 (0.03, 0.10)
0	0.04† (0.01, 0.10)	-0.19 (-0.22, -0.14)	0.24 (0.21, 0.29)	0.27 (0.23, 0.31)	0.07 (0.03, 0.11)	Ambiguous†	Ambiguous†	0	*EA*	-0.06 (-0.14, -0.03)	0	0
0	0.07 (0.03, 0.11)	Ambiguous‡	Ambiguous†	Ambiguous‡	0	0	0.10 (0.05, 0.13)	0.07 (0.,03, 0.11)	Ambiguous†	*C-DEP*	0.25 (0.23, 0.28)	0.16 (0.13, 0.19)
0	Ambiguous†	0	0	0	0	-0.08 (-0.11, -0.02)	Ambiguous†	0.06 (0.02, 0.11)	0.06† (0.01, 0.12)	0.20 (0.18, 0.26)	*ADO-DEP*	0.33 (0.29, 0.35)
0	0	0	0	0	0	Ambiguous†	0	Ambiguous†	-0.07 (-0.12, -0.01)	0.10 (0.04, 0.14)	0.34 (0.28, 0.37)	*ADU-DEP*

Note: values presented represent mean (95% credible interval). Values for the neurodevelopmental variables are from the neurodevelopmental-only model. Green indicates results that were replicated in both TEDS and ALSPAC, orange indicates results that were discordant between TEDS and ALSPAC, and white indicates results that were ambiguous either in TEDS or ALSPAC. † Indicates findings that were ambiguous in the main analysis; ‡ indicates findings that became ambiguous in the sensitivity analysis.

Abbreviations: A = Autistic symptoms; HY = ADHD symptoms – hyperactivity/impulsivity; IN = ADHD symptoms – inattention; GEN COG = general cognitive ability; C-SS = Communication ability – speech & syntax; C-PP = Communication ability – pragmatic; LD-R = Learning ability; EA = Educational achievement; DP = Emotional dysregulation; PP = Peer problems; C-DEP = Childhood depressive symptoms (12 years); ADO-DEP = Adolescent depressive symptoms (16 years); ADU-DEP = Adult depressive symptoms (21 years)

2. Increasing the prior scale (SD = 0.4)

Neurodevelopmental traits.

Nothing relevant.

Neurodevelopmental traits, social-environmental stressors, and co-occurring emotional dysregulation.

In TEDS, academic competence and adult depressive symptoms were classified as ambiguous. In ALSPAC, educational achievement and childhood depressive symptoms were classified as ambiguous.

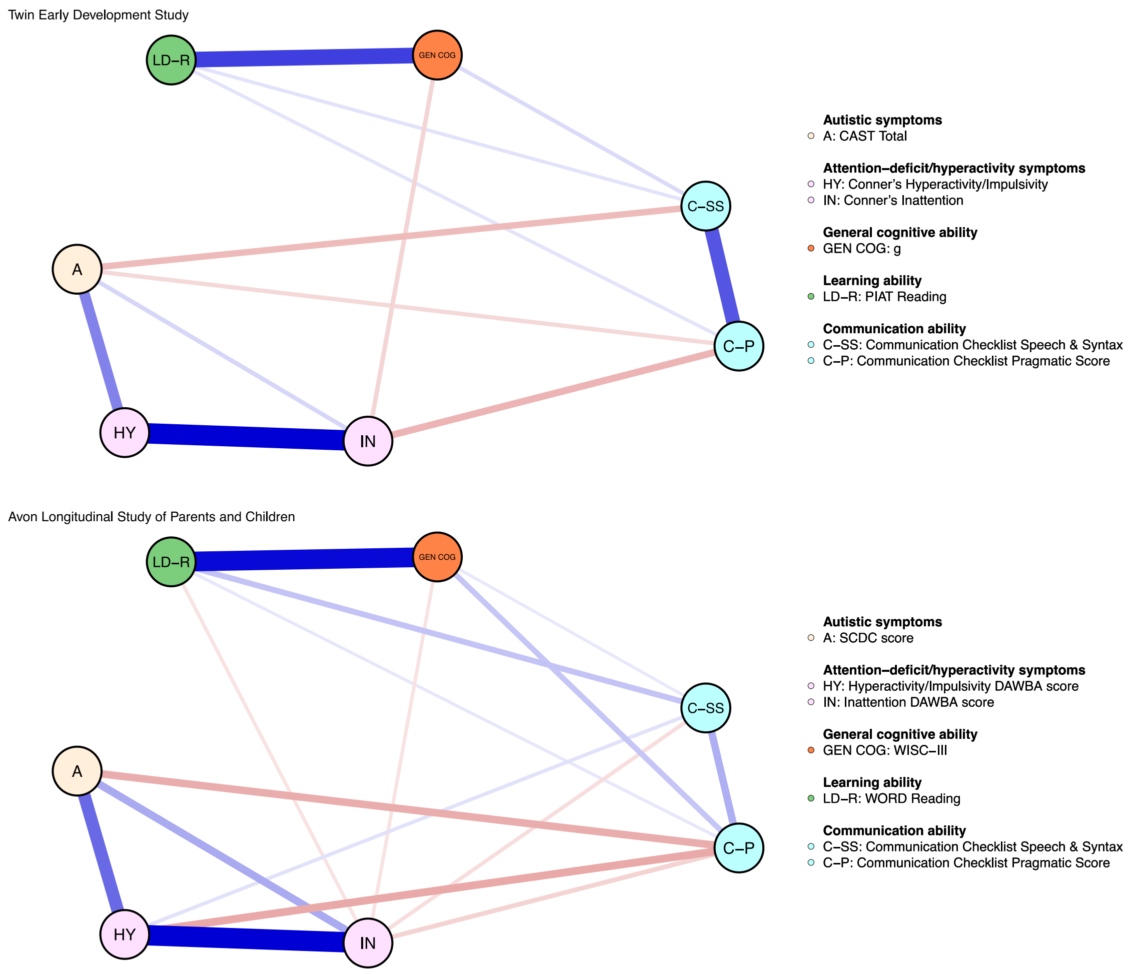

Figure S3. Network plots for neurodevelopmental traits in childhood (7-10 years old) in the Twins Early Development Study (top) and the Avon Longitudinal Study of Parents And Children (bottom) after increasing the prior scale. Variables are represented as nodes (circles) and are colored according to their domain. Edges between two nodes represent partial correlations between two variables. The width of edges is proportional to the strength of the partial correlation. Positive and negative partial correlations were colored in blue and red, respectively.

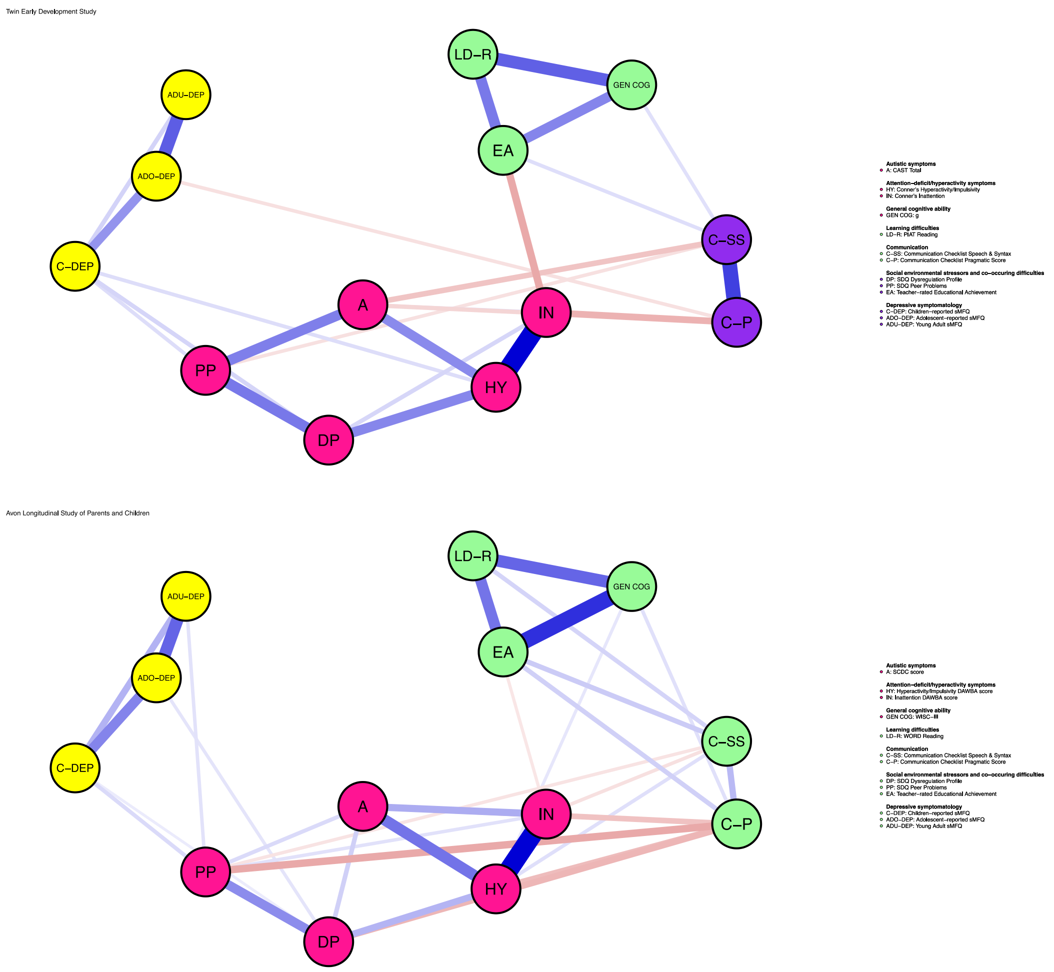

Figure S4. Network plots for neurodevelopmental traits, social-environmental stressors, and co-occurring emotional dysregulation in childhood (7-11 years old) and depressive symptoms over development in childhood (12 years), adolescence (16 years) and adulthood (21 years) in the Twins Early Development Study (top) and the Avon Longitudinal Study of Parents And Children (bottom) after increasing the prior scale. Variables are represented as nodes (circles) and are colored according to their community as identified by the spinglass algorithm. Edges between two nodes represent partial correlations between two variables. The width of edges is proportional to the strength of the partial correlation. Positive and negative partial correlations were colored in blue and red, respectively.

Table S5. Conditional associations between neurodevelopmental traits, social-environmental stressors, co-occurring emotional dysregulation, and depressive symptoms, as well as their relative magnitude, for the Twins Early Development Study (lower triangle) and the Avon Longitudinal Study of Parents And Children (upper triangle) after increasing the prior scale.

A	0.32 (0.30, 0.34)	0.19 (0.16, 0.21)	0	0	0†	-0.18 (-0.20, -0.16)	0.10 (0.07, 0.13)	0.09 (0.06, 0.10)	0	0	0	0
0.29 (0.26, 0.32)	*HY*	0.51 (0.49, 0.53)	0†	0†	0.05 (0.04, 0.09)	-0.18 (-0.21, -0.16)	0.17 (0.14, 0.18)	Ambiguous†	Ambiguous†	0	0	0
0.10 (0.06, 0.13)	0.54 (0.52, 0.57)	*IN*	-0.08 (-0.09, -0.04)	-0.05 (-0.09, -0.04)	-0.06 (-0.1, -0.05)	-0.10 (-0.12, -0.07)	0	0.07 (0.04, 0.09)	0	0	0	0
0	0†	-0.14 (-0.14, -0.06)	*GEN COG*	0.50 (0.47, 0.52)	0.05 (0.03, 0.08)	0.11 (0.10, 0.15)	0	0	0.44 (0.36, 0.46)	Ambiguous†	0†	0
0	0†	0	0.44 (0.40, 0.46)	*LD-R*	0.13 (0.11, 0.16)	0.07 (0.03, 0.08)	0	Ambiguous†	0.26 (0.23, 0.35)	Ambiguous†	Ambiguous†	0
-0.18 (-0.20, -0.12)	0	0†	0.07 (0.05, 0.12)	0.09 (0.03, 0.12)	*C-SS*	0.17 (0.15, 0.20)	0†	-0.06 (-0.08, -0.04)	0.14 (0.03, 0.18)	0	0	Ambiguous†
-0.10 (-0.13, -0.06)	0†	-0.18 (-0.21, -0.14)	0	0.07 (0.03, 0.11)	0.39 (0.35, 0.42)	*C-P*	-0.15 (-0.18, -0.13)	-0.19 (-0.20, -0.16)	0.11 (0.03, 0.07)	0	0	0
Ambiguous†	0.26 (0.21, 0.28)	0.08 (0.05, 0.12)	0	0†	Ambiguous†	0†	*DP*	0.24 (0.22, 0.26)	0	0.06 (0.02, 0.07)	0.04 (0.03, 0.09)	0
0.26 (0.23, 0.29)	0	0†	0	0	-0.06 (-0.10, -0.03)	0	0.26 (0.24, 0.31)	*PP*	0	0.08 (0.05, 0.11)	0	0.09 (0.03, 0.10)
0	Ambiguous†	-0.19 (-0.22, -0.14)	0.24 (0.21, 0.29)	0.27 (0.23, 0.31)	0.07 (0.03, 0.11)	0†	0†	0	*EA*	Ambiguous‡	0	0
0	0.07 (0.03, 0.11)	0	Ambiguous†	0	0	0	0.10 (0.05, 0.13)	0.07 (0.,03, 0.11)	0†	*C-DEP*	0.25 (0.23, 0.28)	0.16 (0.13, 0.19)
0	0†	0	0	0	0	-0.08 (-0.11, -0.02)	0†	Ambiguous‡	Ambiguous†	0.20 (0.18, 0.26)	*ADO-DEP*	0.33 (0.29, 0.35)
0	0	0	0	0	0	0†	0	0†	Ambiguous‡	0.10 (0.04, 0.14)	0.34 (0.28, 0.37)	*ADU-DEP*

Note: values presented represent mean (95% credible interval). Values for the neurodevelopmental variables are from the neurodevelopmental-only model. Green indicates results that were replicated in both TEDS and ALSPAC, orange indicates results that were discordant between TEDS and ALSPAC, and white indicates results that were ambiguous either in TEDS or ALSPAC. † Indicates findings that were ambiguous in the main analysis; ‡ indicates findings that became ambiguous in the sensitivity analysis.

Abbreviations: A = Autistic symptoms; HY = ADHD symptoms – hyperactivity/impulsivity; IN = ADHD symptoms – inattention; GEN COG = general cognitive ability; C-SS = Communication ability – speech & syntax; C-PP = Communication ability – pragmatic; LD-R = Learning ability; EA = Academic competence; PP = Peer problems; DP = Emotional dysregulation; C-DEP = Childhood depressive symptoms (12 years); ADO-DEP = Adolescent depressive symptoms (16 years); ADU-DEP = Adult depressive symptoms (21 years)

3. Complete (≥ 70%) neurodevelopmental data

Summary of changes from the main analyses

Neurodevelopmental traits.

In TEDS, the association between autistic symptoms and general cognitive ability was classified as ambiguous. Intriguingly, some of the associations were discordant in the sensitivity analysis. Specifically, three pairs of variables were found conditionally independent: inattention symptoms and general cognitive ability; learning ability and pragmatic aspects of communication ability; autistic symptoms and pragmatic aspects of communication ability. Additionally, a negative correlation between learning ability and speech & syntax aspects of communication ability was found in the sensitivity analysis.

Neurodevelopmental traits, social-environmental stressors, and co-occurring emotional dysregulation.

In TEDS, academic competence and adult depressive symptoms were classified as ambiguous. In ALSPAC, emotional dysregulation and childhood depressive symptoms were classified as ambiguous.

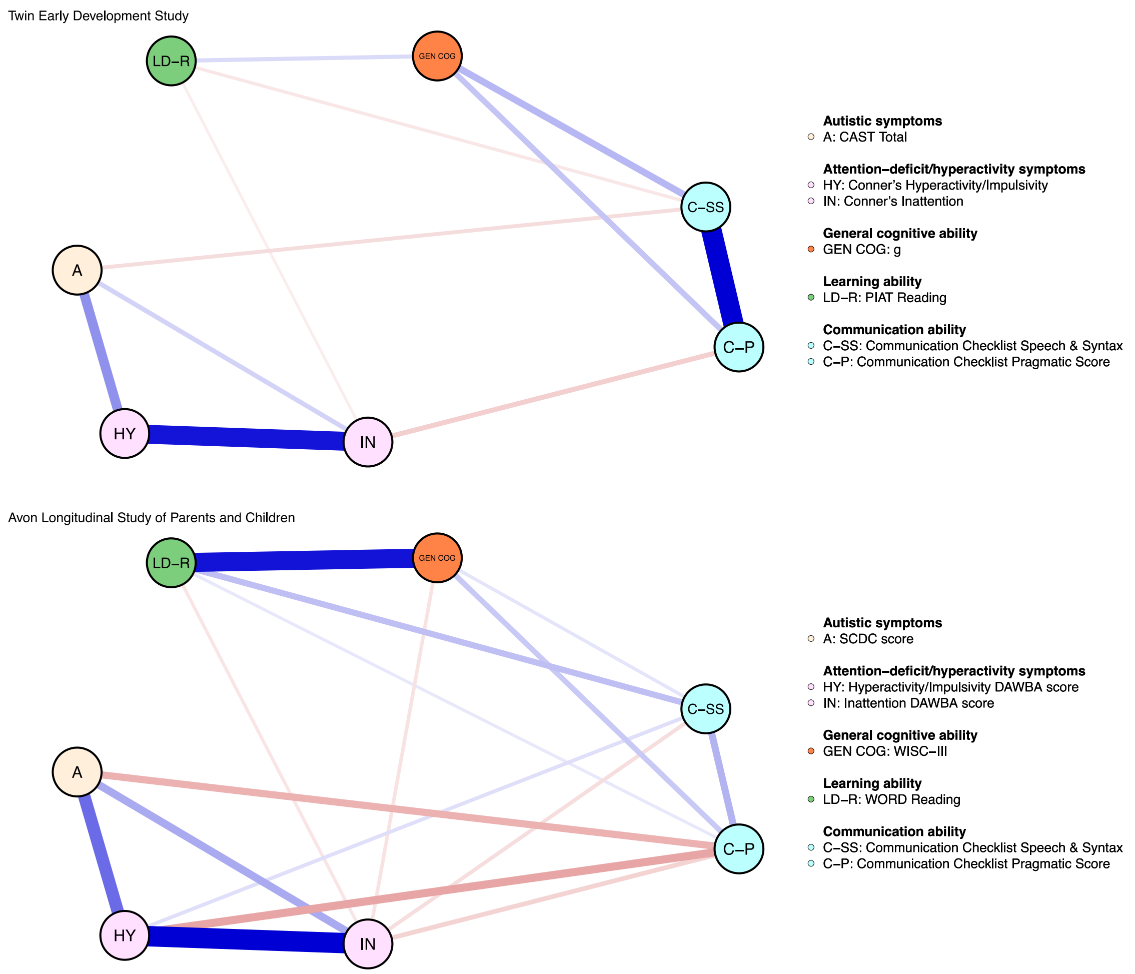

Figure S5. Network plots for neurodevelopmental traits in childhood (7-10 years old) in the Twins Early Development Study (top) and the Avon Longitudinal Study of Parents & Children (bottom) after excluding individuals with > 30% missing neurodevelopmental data. Variables are represented as nodes (circles) and are colored according to their domain. Edges between two nodes represent partial correlations between two variables. The width of edges is proportional to the strength of the partial correlation. Positive and negative partial correlations were colored in blue and red, respectively.

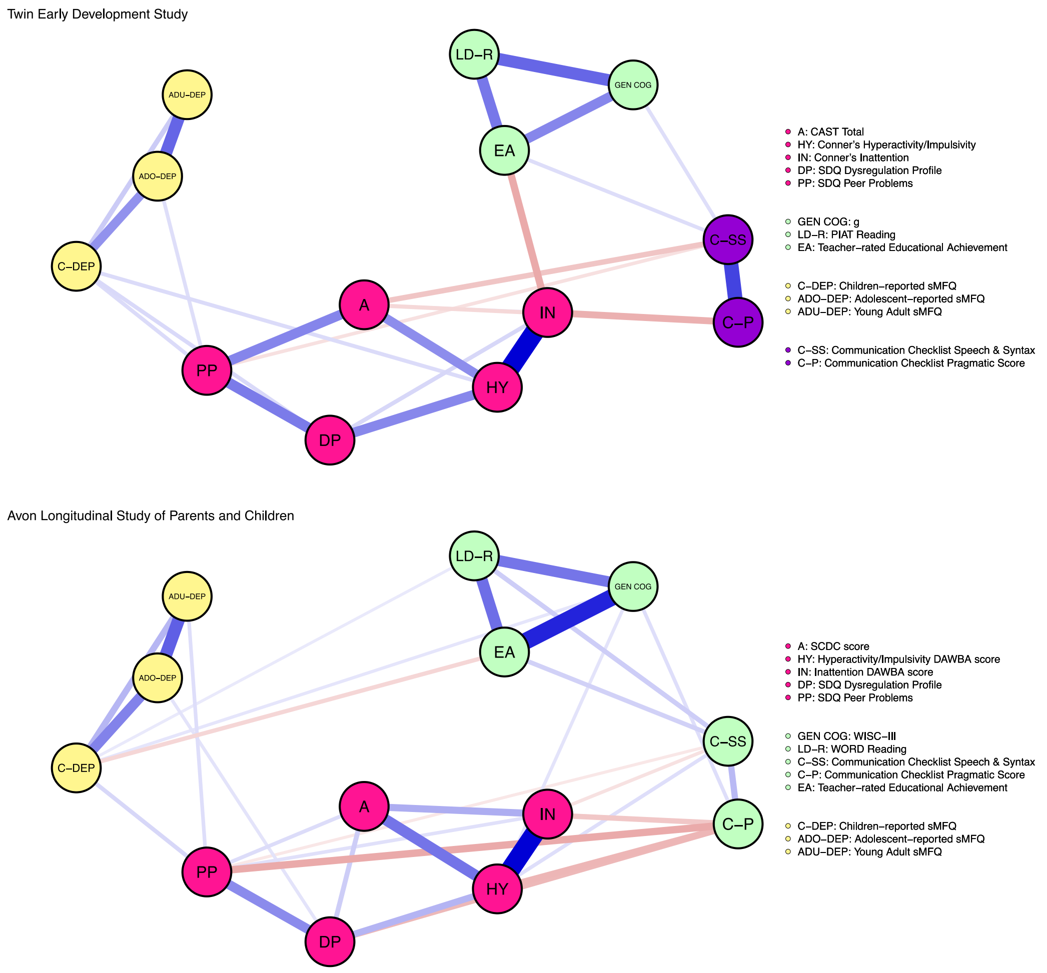

Figure S6. Network plots for neurodevelopmental traits, social-environmental stressors, and co-occurring emotional dysregulation in childhood (7-11 years old) and depressive symptoms over development in childhood (12 years), adolescence (16 years) and adulthood (21 years) in the Twins Early Development Study (top) and the Avon Longitudinal Study of Parents & Children (bottom) after excluding individuals with > 30% missing neurodevelopmental data. Variables are represented as nodes (circles) and are colored according to their community as identified by the spinglass algorithm. Edges between two nodes represent partial correlations between two variables. The width of edges is proportional to the strength of the partial correlation. Positive and negative partial correlations were colored in blue and red, respectively.

Table S6. Conditional associations between neurodevelopmental traits, social-environmental stressors, co-occurring emotional dysregulation, and depression for the Twins Early Development Study (lower triangle) and the Avon Longitudinal Study of Parents & Children (upper triangle) after excluding individuals with > 30% missing neurodevelopmental data.

A	0.31 (0.29, 0.34)	0.19 (0.16, 0.21)	0	0	0†	-0.17 (-0.19, -0.15)	0.09 (0.08, 0.13)	0.09 (0.05, 0.10)	0	0	0	0
0.29 (0.26, 0.32)	*HY*	0.51 (0.49, 0.53)	Ambiguous†	Ambiguous†	0.07 (0.05, 0.1)	-0.20 (-0.22, -0.17)	0.17 (0.13, 0.18)	Ambiguous†	Ambiguous†	0	0	0
0.12 (0.08, 0.15)	0.56 (0.54, 0.59)	*IN*	-0.06 (-0.09, -0.04)	-0.06 (-0.08, -0.03)	-0.06 (-0.1, -0.05)	-0.10 (-0.12, -0.07)	0	0.05 (0.04, 0.09)	Ambiguous‡	0	0	0
Ambiguous‡	Ambiguous†	0§	*GEN COG*	0.48 (0.46, 0.50)	0.05 (0.03, 0.09)	0.11 (0.09, 0.14)	0	0	0.50 (0.39, 0.49)	0.04† (0.02, 0.09)	Ambiguous†	0
0	0†	-0.05§ (-0.09, -0.01)	0.10 (0.06, 0.13)	*LD-R*	0.15 (0.12, 0.17)	0.06 (0.03, 0.08)	0	Ambiguous†	0.25 (0.24, 0.36)	0.03† (0.02, 0.08)	Ambiguous†	0
-0.09 (-0.13, -0.06)	0	0†	0.19 (0.16, 0.23)	-0.07§ (-0.10, -0.03)	*C-SS*	0.16 (0.14, 0.19)	Ambiguous†	-0.04 (-0.08, -0.03)	0.09 (0.04, 0.16)	0	0	Ambiguous†
0§	Ambiguous†	-0.13 (-0.16, -0.10)	0.16§ (0.12, 0.19)	0§	0.60 (0.57, 0.62)	*C-P*	-0.16 (-0.17, -0.13)	-0.18 (-0.20, -0.16)	Ambiguous‡	0	0	0
Ambiguous†	0.24 (0.21, 0.28)	0.08 (0.05, 0.12)	0	0†	Ambiguous†	0†	*DP*	0.24 (0.22, 0.27)	0	Ambiguous‡	0.06 (0.03, 0.09)	0
0.25 (0.22, 0.28)	0	Ambiguous†	0	0	-0.06 (-0.10, -0.03)	0	0.27 (0.24, 0.31)	*PP*	0	0.07 (0.06, 0.11)	0	0.05 (0.03, 0.11)
0	Ambiguous†	-0.18 (-0.22, -0.14)	0.25 (0.20, 0.29)	0.27 (0.23, 0.32)	0.09 (0.03, 0.12)	Ambiguous†	Ambiguous†	0	*EA*	-0.04 (-0.15, -0.03)	0	0
0	0.07 (0.03, 0.12)	0	Ambiguous†	Ambiguous‡	0	0	0.10 (0.05, 0.13)	0.06 (0.,03, 0.12)	0†	*C-DEP*	0.25 (0.23, 0.28)	0.16 (0.13, 0.19)
0	Ambiguous†	0	0	0	0	Ambiguous‡	0†	0.06 (0.02, 0.12)	Ambiguous†	0.25 (0.18, 0.26)	*ADO-DEP*	0.33 (0.29, 0.35)
0	0	0	0	0	0	0†	0	Ambiguous†	Ambiguous‡	0.11 (0.06, 0.16)	0.35 (0.27, 0.36)	*ADU-DEP*

Note: values presented represent mean (95% credible interval). Values for the neurodevelopmental variables are from the neurodevelopmental-only model. Green indicates results that were replicated in both TEDS and ALSPAC, orange indicates results that were discordant between TEDS and ALSPAC, and white indicates results that were ambiguous either in TEDS or ALSPAC. † Indicates findings that were ambiguous in the main analysis; ‡ indicates findings that became ambiguous in the sensitivity analysis; § indicates findings that became discordant in the sensitivity analysis.

Abbreviations: A = Autistic symptoms; HY = ADHD symptoms – hyperactivity/impulsivity; IN = ADHD symptoms – inattention; GEN COG = general cognitive ability; C-SS = Communication ability – speech & syntax; C-PP = Communication ability – pragmatic; LD-R = Learning ability; EA = Academic competence; PP = Peer problems; DP = Emotional dysregulation; C-DEP = Childhood depressive symptoms (12 years); ADO-DEP = Adolescent depressive symptoms (16 years); ADU-DEP = Adult depressive symptoms (21 years)

4. Adjusted for differences in age at data collection and sex
Summary of changes from the main analyses

Neurodevelopmental traits.

In TEDS, the association between autistic symptoms and general cognitive ability was classified as ambiguous. Additionally, the associations between communication abilities and learning ability were also classified as ambiguous. In ALSPAC, the following pairs of variables were classified as ambiguous: autistic symptoms and learning ability; speech & syntax aspects of communication ability and general cognitive ability.

Neurodevelopmental traits, social-environmental stressors, and co-occurring emotional dysregulation.

In TEDS, the following pairs of variables were classified as ambiguous: inattention symptoms and adolescent depressive symptoms; speech & syntax communication ability and peer problems; academic competence and adult depressive symptoms. In ALSPAC, autistic symptoms, emotional dysregulation, academic performance and childhood depressive symptoms were classified as ambiguous.

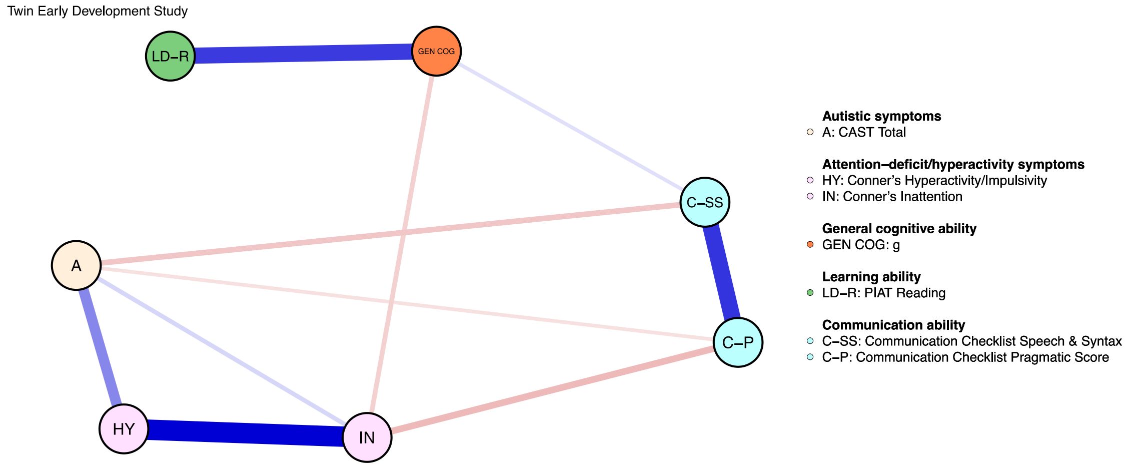

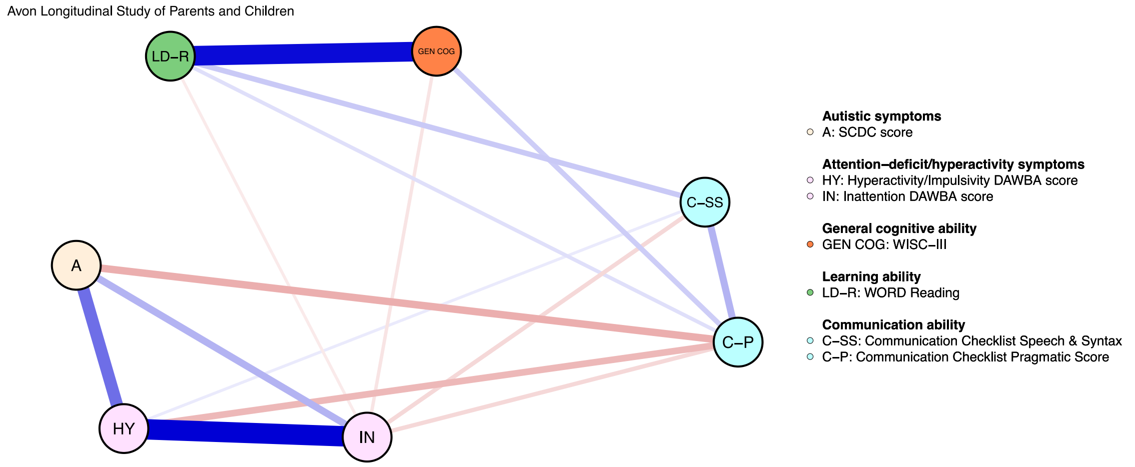

Figure S7. Network plots for neurodevelopmental traits in childhood (7-10 years old) in the Twins Early Development Study (top) and the Avon Longitudinal Study of Parents & Children (bottom) after adjusting for age at data collection and sex. Variables are represented as nodes (circles) and are colored according to their domain. Edges between two nodes represent partial correlations between two variables. The width of edges is proportional to the strength of the partial correlation. Positive and negative partial correlations were colored in blue and red, respectively.

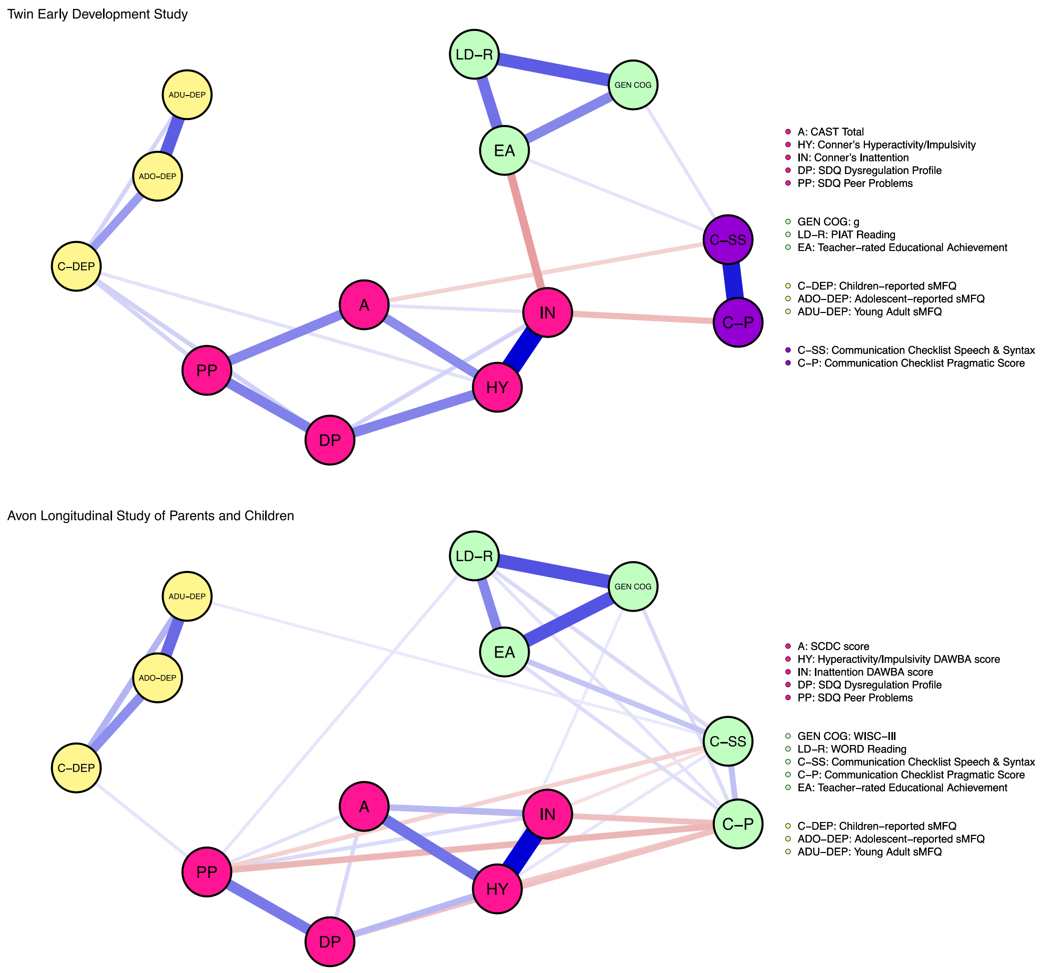

Figure S8. Network plots for neurodevelopmental traits, social-environmental stressors, and co-occurring emotional dysregulation in childhood (7-11 years old) and depressive symptoms over development in childhood (12 years), adolescence (16 years) and adulthood (21 years) in the Twins Early Development Study (top) and the Avon Longitudinal Study of Parents & Children (bottom) after adjusting for age at data collection and sex. Variables are represented as nodes (circles) and are colored according to their community as identified by the spinglass algorithm. Edges between two nodes represent partial correlations between two variables. The width of edges is proportional to the strength of the partial correlation. Positive and negative partial correlations were colored in blue and red, respectively.

Table S7. Conditional associations between neurodevelopmental traits, social-environmental stressors, co-occurring emotional dysregulation, and depression for the Twins Early Development Study (lower triangle) and the Avon Longitudinal Study of Parents & Children (upper triangle) after adjusting for sex and age at data collection.

A	0.32 (0.30, 0.34)	0.17 (0.15, 0.20)	0	Ambiguous‡	Ambiguous†	-0.19 (-0.21, -0.16)	0.07 (0.06, 0.13)	0.06 (0.04, 0.09)	0	Ambiguous‡	0	0
0.27 (0.25, 0.31)	HY	0.53 (0.51, 0.55)	Ambiguous†	0†	0.05 (0.04, 0.09)	-0.16 (-0.19, -0.14)	0.17 (0.13, 0.19)	0†	Ambiguous†	0	0	0
0.10 (0.06, 0.13)	0.53 (0.51, 0.56)	IN	-0.08 (-0.09, -0.04)	-0.05 (-0.07, -0.03)	-0.06 (-0.1, -0.05)	-0.09 (-0.11, -0.07)	0	0.08 (0.06, 0.11)	Ambiguous‡	0	0	0
Ambiguous‡	0†	-0.11 (-0.15, -0.07)	GEN COG	0.51 (0.50, 0.53)	Ambiguous‡	0.12 (0.09, 0.14)	0	0	0.35 (0.30, 0.42)	Ambiguous†	0†	0
0	0†	Ambiguous‡	0.43 (0.39, 0.46)	LD-R	0.12 (0.10, 0.15)	0.07 (0.05, 0.10)	0	0.08† (0.02, 0.09)	0.26 (0.20, 0.32)	0†	0†	0
-0.16 (-0.17, -0.09)	0	Ambiguous†	0.08 (0.04, 0.11)	Ambiguous‡	C-SS	0.18 (0.15, 0.20)	0†	-0.1 (-0.13, -0.07)	0.14 (0.03, 0.18)	0	0	0.05† (0.01, 0.08)
-0.08 (-0.12, -0.03)	0†	-0.13 (-0.21, -0.11)	0	Ambiguous‡	0.31 (0.29, 0.52)	C-P	-0.14 (-0.16, -0.11)	-0.16 (-0.19, -0.14)	0.11 (0.03, 0.07)	0	Ambiguous‡	Ambiguous‡
0†	0.26 (0.21, 0.29)	0.08 (0.05, 0.13)	0	0†	Ambiguous†	Ambiguous†	DP	0.28 (0.26, 0.31)	0	Ambiguous‡	Ambiguous‡	0
0.26 (0.20, 0.28)	0	Ambiguous†	0	0	Ambiguous‡	0	0.23 (0.18, 0.33)	PP	0	0.05 (0.03, 0.09)	0	Ambiguous‡
0	Ambiguous†	-0.22 (-0.24, -0.16)	0.20 (0.20, 0.28)	0.29 (0.24, 0.32)	0.07 (0.02, 0.10)	0†	0†	0	EA	Ambiguous‡	0	0
0	0.03 (0.02, 0.10)	0	Ambiguous†	Ambiguous‡	0	0	0.11 (0.05, 0.14)	0.08 (0.,03, 0.14)	Ambiguous†	C-DEP	0.27 (0.23, 0.29)	0.16 (0.12, 0.19)
0	0†	Ambiguous‡	0	0	0	0§	Ambiguous†	0§	Ambiguous†	0.19 (0.16, 0.25)	ADO-DEP	0.30 (0.29, 0.36)
0	0	0	0	0	0	Ambiguous†	0	Ambiguous†	Ambiguous‡	0.10 (0.04, 0.13)	0.33 (0.28, 0.36)	ADU-DEP

Note: values presented represent mean (95% credible interval). Values for the neurodevelopmental variables are from the neurodevelopmental-only model. Green indicates results that were replicated in both TEDS and ALSPAC, orange indicates results that were discordant between TEDS and ALSPAC, and white indicates results that were ambiguous either in TEDS or ALSPAC. † Indicates findings that were ambiguous in the main analysis; ‡ indicates findings that became ambiguous in the sensitivity analysis; § indicates findings that became discordant in the sensitivity analysis.

Abbreviations: A = Autistic symptoms; HY = ADHD symptoms – hyperactivity/impulsivity; IN = ADHD symptoms – inattention; GEN COG = general cognitive ability; C-SS = Communication ability – speech & syntax; C-PP = Communication ability – pragmatic; LD-R = Learning ability; EA = Educational Achievement; PP = Peer problems; DP = Emotional dysregulation; C-DEP = Childhood depressive symptoms (12 years); ADO-DEP = Adolescent depressive symptoms (16 years); ADU-DEP = Adult depressive symptoms (21 years)

REFERENCES

1. Ronald A, Happé F, Bolton P, et al. Genetic heterogeneity between the three components of the autism spectrum: a twin study. J Am Acad Child Adolesc Psychiatry. 2006;45:691-699.
2. Ronald A, Happé F, Price TS, et al. Phenotypic and genetic overlap between autistic traits at the extremes of the general population. J Am Acad Child Adolesc Psychiatry. 2006;45:1206-1214.
3. Borsboom D, Deserno MK, Rhemtulla M, et al. Network analysis of multivariate data in psychological science. Nature Reviews Methods Primers. 2021;1:58.
4. Fried EI, von Stockert S, Haslbeck JMB, et al. Using network analysis to examine links between individual depressive symptoms, inflammatory markers, and covariates. Psychol Med. 2020;50:2682-2690.
5. Williams DR, Briganti G, Linkowski P, et al. On Accepting the Null Hypothesis of Conditional Independence in Partial Correlation Networks: A Bayesian Analysis. PsyArXiv Preprints. 2021.
6. Williams DR, Mulder J. Bayesian hypothesis testing for Gaussian graphical models: Conditional independence and order constraints. Journal of Mathematical Psychology. 2020;99:102441.
7. Williams DR. Learning to live with sampling variability: Expected replicability in partial correlation networks. Psychol Methods. 2022.
8. Eyre O, Hughes RA, Thapar AK, et al. Childhood neurodevelopmental difficulties and risk of adolescent depression: the role of irritability. J Child Psychol Psychiatry. 2019;60:866-874.
9. de Ron J, Fried EI, Epskamp S. Psychological networks in clinical populations: investigating the consequences of Berkson's bias. Psychol Med. 2021;51:168-176.
10. Rimfeld K, Malanchini M, Spargo T, et al. Twins Early Development Study: A Genetically Sensitive Investigation into Behavioral and Cognitive Development from Infancy to Emerging Adulthood. Twin Res Hum Genet. 2019;22:508-513.
11. Boyd A, Golding J, Macleod J, et al. Cohort Profile: the 'children of the 90s'--the index offspring of the Avon Longitudinal Study of Parents and Children. Int J Epidemiol. 2013;42:111-127.
12. Fraser A, Macdonald-Wallis C, Tilling K, et al. Cohort Profile: the Avon Longitudinal Study of Parents and Children: ALSPAC mothers cohort. Int J Epidemiol. 2013;42:97-110.
13. Northstone K, Lewcock M, Groom A, et al. The Avon Longitudinal Study of Parents and Children (ALSPAC): an update on the enrolled sample of index children in 2019. Wellcome Open Res. 2019;4:51.
14. Scott FJ, Baron-Cohen S, Bolton P, et al. The CAST (Childhood Asperger Syndrome Test): preliminary development of a UK screen for mainstream primary-school-age children. Autism. 2002;6:9-31.
15. Williams J, Scott F, Stott C, et al. The CAST (Childhood Asperger Syndrome Test): test accuracy. Autism. 2005;9:45-68.
16. Williams J, Allison C, Scott F, et al. The Childhood Asperger Syndrome Test (CAST): test-retest reliability. Autism. 2006;10:415-427.
17. Skuse DH, Mandy WPL, Scourfield J. Measuring autistic traits: heritability, reliability and validity of the Social and Communication Disorders Checklist. British Journal of Psychiatry. 2005;187:568-572.
18. Bölte S, Westerwald E, Holtmann M, et al. Autistic traits and autism spectrum disorders: the clinical validity of two measures presuming a continuum of social communication skills. J Autism Dev Disord. 2011;41:66-72.
19. Conners CK, Sitarenios G, Parker JD, et al. The revised Conners' Parent Rating Scale (CPRS-R): factor structure, reliability, and criterion validity. J Abnorm Child Psychol. 1998;26:257-268.
20. Goodman R, Ford T, Richards H, et al. The Development and Well-Being Assessment: description and initial validation of an integrated assessment of child and adolescent psychopathology. J Child Psychol Psychiatry. 2000;41:645-655.
21. Wechsler D. Wechsler intelligence scale for children - Third Edition UK (WISC-III-UK) Manual. The Psychological Corporation, London. 1992.
22. Smith P, Fernandes C, Strand S. Cognitive abilities test 3 (CAT3). nferNelson, Windsor. 2001.
23. Bishop DV. Development of the Children's Communication Checklist (CCC): a method for assessing qualitative aspects of communicative impairment in children. J Child Psychol Psychiatry. 1998;39:879-891.
24. Haworth CM, Harlaar N, Kovas Y, et al. Internet cognitive testing of large samples needed in genetic research. Twin Res Hum Genet. 2007;10:554-563.
25. Wechsler D. Wechsler Objective Reading Dimensions. The Psychological Corporation, London. 1993.
26. Goodman R. The Strengths and Difficulties Questionnaire: a research note. J Child Psychol Psychiatry. 1997;38:581-586.
27. Haworth CM, Kovas Y, Petrill SA, et al. Developmental origins of low mathematics performance and normal variation in twins from 7 to 9 years. Twin Res Hum Genet. 2007;10:106-117.
28. Holtmann M, Becker A, Banaschewski T, et al. Psychometric validity of the strengths and difficulties questionnaire-dysregulation profile. Psychopathology. 2011;44:53-59.

29. Holtmann M, Buchmann AF, Esser G, et al. The Child Behavior Checklist-Dysregulation Profile predicts substance use, suicidality, and functional impairment: a longitudinal analysis. J Child Psychol Psychiatry. 2011;52:139-147.
30. Deutz MHF, Shi Q, Vossen HGM, et al. Evaluation of the Strengths and Difficulties Questionnaire-Dysregulation Profile (SDQ-DP). Psychol Assess. 2018;30:1174-1185.
31. Angold A, Costello EJ. Mood and Feelings Questionnaire (MFQ). Developmental Epidemiology Program, Durham, NC. 1987.
32. Angold A, Costello EJ, Messer SC, et al.: Development of a short questionnaire for use in epidemiological studies of depression in children and adolescents. US, John Wiley & Sons; 1995. pp. 237-249.
33. Eyre O, Bevan Jones R, Agha SS, et al. Validation of the short Mood and Feelings Questionnaire in young adulthood. Journal of Affective Disorders. 2021;294:883-888.
34. Preacher KJ, Kelley K. Effect size measures for mediation models: quantitative strategies for communicating indirect effects. Psychol Methods. 2011;16:93-115.
